# ﻿A new genus and species of oceanic planktonic Tisbidae (Crustacea, Copepoda, Harpacticoida) with enlarged modified eyes

**DOI:** 10.3897/zookeys.1191.114974

**Published:** 2024-02-15

**Authors:** Sota Komeda, Susumu Ohtsuka, Rony Huys

**Affiliations:** 1 Marine Ecology Research Institute, 300 Iwawada, Onjuku-machi, Chiba Prefecture 299-5105, Japan Marine Ecology Research Institute Chiba Japan; 2 Fisheries Laboratory, Blue Innovation Division, Setouchi Carbon Neutral Research Center, Hiroshima University, 5-8-1 Minato-machi, Takehara City, Hiroshima Prefecture 725-0024, Japan Hiroshima University Hiroshima Japan; 3 Science Group, The Natural History Museum, London SW7 5BD, UK The Natural History Museum London United Kingdom

**Keywords:** Caudal ramus, ecological radiation, Gicklhorn’s organ, key to species, Kuroshio, mesopelagic, taxonomy, zooplankton

## Abstract

Both sexes of a new monotypic genus of Tisbidae (Copepoda, Harpacticoida) are described from the epi- or mesopelagic zone in the Kuroshio region, Japan. *Gyoromeguttatum***gen. et sp. nov.** belongs to a monophyletic lineage of deepwater holoplanktonic genera defined by a suite of characters. Within this clade, *Gyorome***gen. nov.** appears most closely related to *Neotisbella* Boxshall, 1979. The most distinguishable feature of *G.guttatum***gen. et sp. nov.** is the presence of large, paired, frontal modified eyes, each consisting of a baculiform ocellus, a globular (Gicklhorn’s?) organ, and a semi-parabolic plate. The taxonomic position of *Tisbespinulosa* Bradford & Wells, 1983 is discussed and a key to the six meso- and bathypelagic tisbid species is provided. Confusion surrounding earlier literature reports of supernumerary elements on the caudal ramus in some harpacticoid taxa is clarified. Secondary modifications of ocellar components of the typical naupliar eye in the Harpacticoida are reviewed. It is suggested that the development of specialized eyes in *G.guttatum***gen. et sp. nov.** may provide a means for detecting bioluminescent food particles in oligotrophic pelagic environments. The large, vaulted prosome indicates the species is an opportunistic macrophage that has adopted gorging as a feeding strategy.

## ﻿Introduction

Lang’s (1944, 1948) revision of the family Tisbidae Stebbing, 1910 (Crustacea, Copepoda, Harpacticoida) assigned the 12 genera recognized at the time to two subfamilies. In the Tisbinae he placed *Tisbe* Lilljeborg, 1853 (type genus), *Scutellidium* Claus, 1866, *Cholidya* Farran, 1914, *Sacodiscus* Wilson, 1924, and *Tisbella* Gurney, 1927. The new subfamily Idyanthinae was proposed by Lang (1944) to accommodate *Zosime* Boeck, 1873, *Idyella* Sars, 1905, *Idyanthe* Sars, 1909a, *Tachidiella* Sars, 1909b, *Pseudozosime* Scott, 1912 and *Idyellopsis* Lang, 1948, in addition to *Tachidiopsis* Sars, 1911 which was classified as *incertae sedis*. A third subfamily, the Cholidyinae, was proposed by Boxshall (1979) for *Cholidya*, a parasite of cephalopods (Humes and Voight 1997), but no proper justification was provided for this course of action.

The Idyanthinae was raised to family rank by Seifried (2003) to which the following genera have been added since: *Dactylopia* Becker, 1974; *Styracothorax* Huys, 1993; *Aspinothorax* Moura & Martínez Arbizu, 2003; *Meteorina* George, 2004; *Nematovorax* Bröhldick, 2005 and *Pseudometeorina* George & Wiest, 2015 (as genus incertae sedis but see George 2023). A new family, the Zosimeidae [for correct spelling see Huys and Clark (2009) and Anonymous (2010)], was established to accommodate *Zosime*, *Pseudozosime* and *Peresime* Dinet, 1974 while *Tachidiopsis* was transferred to the Neobradyidae (Seifried 2003).

The Tisbinae saw the addition of *Paraidya* Sewell, 1940 (an unavailable name subsequently validated by Huys (2009) under his authorship) and *Tisbintra* Sewell, 1940, both genera were not considered by Lang (1944, 1948), and *Neoscutellidium* Zwerner, 1967. Boxshall (1979) discussed the relationships between the tisbinid genera, reinstated *Bathyidya* Farran, 1926 (previously a junior subjective synonym of *Tisbe*; see also Volkmann 1979b), and added two new genera, *Neotisbella* Boxshall, 1979 and *Volkmannia* Boxshall, 1979. Itô (1976) had previously reinstated *Scutellopsis* Wiborg, 1964 from the synonymy of *Scutellidium* while Dahms & Dieckmann, (1987) proposed *Drescheriella* Dahms & Dieckmann, 1987 as a new addition to the Tisbinae. Moura and Martínez Arbizu (2003) postulated that the family Porcellidiidae is nested within the Tisbidae, most likely as the sistergroup of *Sacodiscus*, but this hypothesis did not gain any acceptance (Wells 2007).

Following Boxshall’s (1979) proposal of the Cholidyinae, Avdeev (1982, 1983, 1986) subsequently described five new genera associated with deep water octopodans but created taxonomic confusion by placing three of them in the Cholidyinae (*Cholidyella* Avdeev, 1982; *Brescianiana* Avdeev, 1982; *Tripartisoma* Avdeev, 1983) and the remaining two in the Tisbinae (*Yunona* Avdeev, 1983; *Octopinella* Avdeev, 1986). This subfamilial assignment, effectively implying a dual colonization of cephalopod mollusks by two sister lineages, was uncritically adopted by most authors (Bresciani and Lützen 1994; Humes and Voight 1997; López-González et al. 2000; Wells 2007) while the more parsimonious alternative involving a single colonization event was favored by Huys (2016) who also considered *Neoscutellidium* (parasitic on fish) a member of the same monophyletic lineage. In this scenario, the Tisbinae, as currently defined, constitute a paraphyletic group at the exclusion of the Cholidyinae, implying that the current subfamilial division of the Tisbidae is meaningless and must be abandoned. With the addition of *Avdeevia* Bresciani & Lützen, 1994, *Genesis* López-González, Bresciani & Huys in López-González et al. 2000 and *Amplipedicola* Avdeev, 2010 (all of which parasitize cephalopod hosts) the current number of genera in the Tisbidae stands at 21.

Members of the family Tisbidae exhibit a variety of lifestyles ranging from free-living to obligatory parasitic. Although all species are exclusively marine, the family as a whole serves as a typical example illustrating the complex ecological radiation that characterizes the evolutionary history of harpacticoid copepods. Tisbids, in particular species of the genera *Tisbe* and *Scutellidium*, show a universal occurrence of parallelism in phytal habitats (Hicks 1980, 1985), either as associates of the sediments trapped by algae when the fronds and holdfasts are heavily loaded with silt-clay or detritus, or as genuine algae-dwelling forms (Hicks and Coull 1983). In most cases a critical experimental verification of their trophic dependence on the algae does not exist. Others, such as members of *Tisbintra* and *Tisbella* are commonly found in surface plankton samples or mangrove ecosystems (Willey 1930; Sewell 1940; Ummerkutty 1961; Volkmann 1979a; Gómez and Fuentes-Reines 2017; Fuentes-Reinés and Suárez Morales 2019) and coastal marine (Coull 1970; Coull and Herman 1970; Fleeger and Shirley 1990; Franz and Friedman 2002) and brackish water habitats (Gurney 1927; Wilson 1932; Yeatman 1963, 1983; Reid and Hribar 2006; Morales-Serna and Gómez 2008). An increasing volume of literature has demonstrated that members of *Drescheriella* are sympagic (sea-ice inhabiting) and are often associated with microalgae colonizing the cracks in the sea ice (e.g., Giesbrecht 1902; Dahms and Dieckmann 1987; Dahms et al. 1990; Dahms and Schminke 1992; Schnack-Schiel et al. 1998, 2001a, b, 2004, 2008; Swadling et al. 2000; Kiko et al. 2008; Loots et al. 2009; Kramer et al. 2011; Wallis et al. 2016; Makabe et al. 2022).

In the Tisbidae, twenty-five species have entered into symbiotic associations with metazoan hosts (mollusks, echinoderms, crustaceans and teleost fish), representing ten independent colonization events (Huys 2016). Three of those events involve mollusk hosts, including cephalopods, bivalves, and gastropods. Most members of the “Cholidyinae” utilize deep water octopuses as hosts and complete the entire copepodid phase inside the tissues of the cephalopod while the free-swimming phase is presumably reduced to the naupliar and adult stages (López-González et al. 2000). Two species of *Tisbe* have been reported from the mantle cavity of mussels (*Mytilus* spp.) in both North and South America (Humes 1954; Huys and Song 2004; Cremonte et al. 2015; Huys 2016), representing the only records of tisbids associated with bivalved mollusks. The only association between marine gastropods and harpacticoid copepods was reported by Branch (1974) who found large numbers of all developmental stages of *Scutellidiumpatellarum* Branch, 1974, in the pallial cavity of five species of *Patella* L. in South Africa. Huys (2016) documented four independent associations between tisbids and crustacean hosts. *Sacodiscusovalis* (Wilson, 1944) lives as an ectosymbiont on the exoskeleton of the American lobster *Homarusamericanus* H. Milne Edwards, 1837 in North America (Wilson 1944; Humes 1960). *Tisbeelongata* (A. Scott, 1896) spends most of its life cycle in the gill chamber of the European lobster *Homarusgammarus* (Linnaeus, 1758) in British waters (Gooding 1957; Bruce et al. 1963; Holmes and O’Connor 1990; Gotto 1993; Gurney 1933). An undescribed species of *Tisbe* was recorded from the gills of the red king crab, *Paralithodescamtschaticus* (Tilesius, 1815), in the Barents Sea (Haugen et al. 1998; Jansen et al. 1998; Dvoretsky and Dvoretsky 2013, 2023). The three known species of *Paraidya* are exclusively associated with Indo-Pacific diogenid anomuran crabs of the genus *Dardanus* Paul’son, 1875 (Humes and Ho 1969; Humes 1981; Innocenti 2009). Two tisbid species are known to live in associations with echinoderm hosts (Huys 2016). *Tisbejaponica* Ho, 1982 is an associate of the blue bat star *Patiriapectinifera* (Müller & Troschel, 1842) in the Sea of Japan and the only harpacticoid known to utilize starfish hosts (Ho 1982). Stock (1960) recovered *Sacodiscushumesi* Stock, 1960 from washings of Holothuria (Holothuria) tubulosa Gmelin, 1791 collected in the Bay of Banyuls, France but this association requires confirmation (Huys 2016). Finally, *Neoscutellidiumyeatmani* Zwerner, 1967 occurs on the gills of the bathydemersal Antarctic eelpout, *Lycodichthysdearborni* (DeWitt, 1962), and is the only confirmed record of a harpacticoid utilizing a fish host (Zwerner 1967).

Only few harpacticoid families have secondarily colonized open oceanic waters (Boxshall 1979; Huys and Böttger-Schnack 1994; Huys and Conroy-Dalton 2000) and their evolutionary success in terms of diversification in the oceanic realm has generally remained limited. The Tisbidae contains a monophyletic clade uniting three genera (*Bathyidia*, *Neotisbella*, *Volkmannia*) that are exclusively found in the meso- and bathypelagic zones of the Atlantic Ocean (Farran 1926; Deevey and Brooks 1977; Boxshall 1979; Khodami et al. 2017). Here we describe a new genus and species of oceanic planktonic Tisbidae from the epi- or mesopelagic zone of the Kuroshio region, Japan, compare its unique morphological features and discuss its relationships with other deepwater genera in the family.

## ﻿Material and methods

The copepods were collected in the Kuroshio region, Japan (33°10'N, 136°00'E) in the daytime (1423–1650) on 28 November 2018 during the 1828 research cruise by the TRV SEISUI-MARU of Mie University. An oblique tow (sampling depth 0–935 m) at speed of 2 knots was performed using an ORI net (diameter 160 cm, mesh size 330 µm; cf. Omori 1965). Specimens were fixed in 10% neutralized formalin seawater immediately after capture, cleared in lactophenol, and dissected under an Olympus SZX stereo microscope. Illustrations were drawn using an Olympus BX53 compound microscope equipped with a drawing tube. The descriptive terminology is adopted from Huys and Boxshall (1991) and Huys et al. (1996). Abbreviations used in the text are ae, aesthetasc; P1–P6, for legs 1–6; exp, enp for exopod and endopod, respectively; exp (enp)-1 (-2, -3) to denote the proximal (middle, distal) segments of a ramus. Type specimens (NMST-Cr 31562–31565) were deposited in the National Museum of Nature and Science (NSMT; Tsukuba, Ibaraki Prefecture, Japan).

## ﻿Systematics

### ﻿Order Harpacticoida Sars, 1903

**Family Tisbidae Stebbing, 1910**

#### ﻿Genus Gyorome gen. nov.

Taxon classificationAnimaliaHarpacticoidaTisbidae

6E9B0BE2-D07B-5599-8FB4-28D68E728805

https://zoobank.org/D0C2FE57-C793-4EEA-A36A-609F05494255

##### Diagnosis.

Tisbidae. Body cyclopiform, large (> 1 mm); genital and first abdominal somites completely fused in ♀, forming genital double-somite. Sexual dimorphism in prosomal ornamentation, antennule, maxilliped, P2 endopod, P5, P6 and urosomal segmentation. Prosome capacious and vaulted; dorsal surface pustulate (covered by dense pattern of denticles); posterior margin of cephalothorax with middorsal protrusion in ♀, absent in ♂. Cephalic region with large, paired, modified eyes, each comprising a baculiform ocellus, a globular (Gicklhorn’s?) organ and a semi-parabolic plate. Caudal ramus with seven setae and paired multi-branched tube-pores along posterior margin, displacing setae III–VI towards inner distal corner.

Antennule short, relatively compact and 8-segmented in ♀, with aesthetasc on segment 4; slender, 8-segmented and haplocer in ♂, with geniculation between segments 6 and 7, and aesthetasc on segment 4 and elongate digitiform segment 8. Antenna without seta on basis and proximal endopodal segment; exopod 4-segmented with armature [2, 1, 1, 3]. Mandible with unarmed basis and 1-segmented rami; exopod with one lateral and two terminal setae; endopod with two lateral and four terminal setae. Maxillule 3-segmented, comprising praecoxa, endopod, and compound segment representing fused coxa, basis and exopod. Maxilla 2-segmented, comprising syncoxa and allobasis; syncoxa with small coxal endite bearing one plumose seta; allobasis produced into curved claw with fine pinnules along outer margin and short plumose seta just over halfway claw length. Maxilliped ♀ 3-segmented, comprising short syncoxa articulating with subcylindrical pedestal, unarmed elongate basis, and small endopod with one unipinnate lateral seta and long, slender, distal claw accompanied at base by plumose seta. Maxilliped ♂ with modified basis (distal palmar margin produced into lobate spinular expansion) and endopod (with unguiform projection along medial margin).

P1–P4 with 3-segmented exopods and endopods; with dense pattern of minute spinules on anterior surface of protopod and rami. P1 outer spines on exp-2 and -3 without spinular combs; exp-2 not markedly longer than other exopodal segments. P1 endopod non-prehensile; indistinctly 3-segmented with transverse surface suture marking original segmentation between enp-2 and -3; enp-1 expanded in distal half forming lobate extension along medial margin; enp-3 small. P2 enp-1 inner seta modified in ♂, displaying pinnate ornamentation along distal half of outer margin (instead of plumose in ♀). Armature formula of P1–P4 as follows (Roman and Arabic numerals indicate spines and setae, respectively):

**Table:** 

	Coxa	Basis	Exopod			Endopod		
			1	2	3	1	2	3
Leg 1 (P1)	0–0	I–I	I–0	I–1	I+5	0–1	0–1	3
Leg 2 (P2)	0–0	1–0	I–1	I–1	III, I+1, 2	0–1	0–2	I, 2, 2
Leg 3 (P3)	0–0	1–0	I–1	I–1	III, I+1, 3	0–1	0–2	I, 2, 3
Leg 4 (P4)	0–0	1–0	I–1	I–1	III, I+1, 3	0–1	0–2	I, 2, 2

P5 2-segmented in both sexes, comprising baseoendopod and elongate exopod; obsolete endopodal lobe represented by one seta (minute in ♂); exopod with one outer, one inner and two terminal well developed setae. P6 with two minute setae in ♀; sixth pair of legs symmetrical in ♂, each with three well developed setae.

##### Type species.

*Gyoromeguttatum* gen. et sp. nov. (by original designation).

##### Etymology.

The generic name is based on the Japanese word “Gyorome”, meaning “bulging eyes” and refers to the large ocelli in the cephalosome of the type species. Gender neuter.

#### ﻿ Gyorome guttatum

Taxon classificationAnimaliaHarpacticoidaTisbidae

gen. et sp. nov.

7BD58734-0C72-5157-8706-D90C23AD9EEC

https://zoobank.org/20C65F2B-C974-40F9-B6E8-E7167EB00802

**Figure 1.**
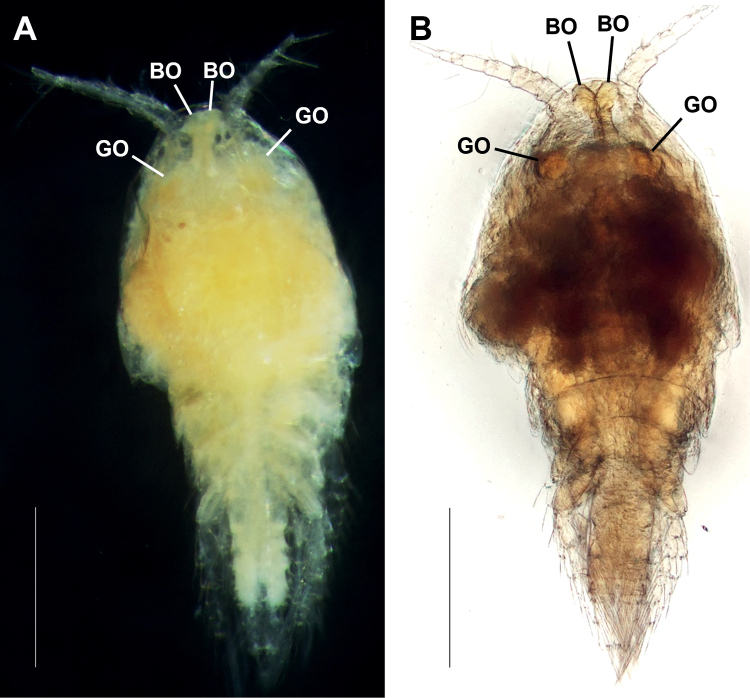
Focus stacked micrographs of *Gyoromeguttatum* gen. et sp. nov., adult female, holotype, using **A** reflected (incident) light microscopy and **B** transmitted light microscopy. Abbreviations: BO, baculiform ocelli; GO, globular organs. Scale bars: 0.5 mm.

**Figure 2.**
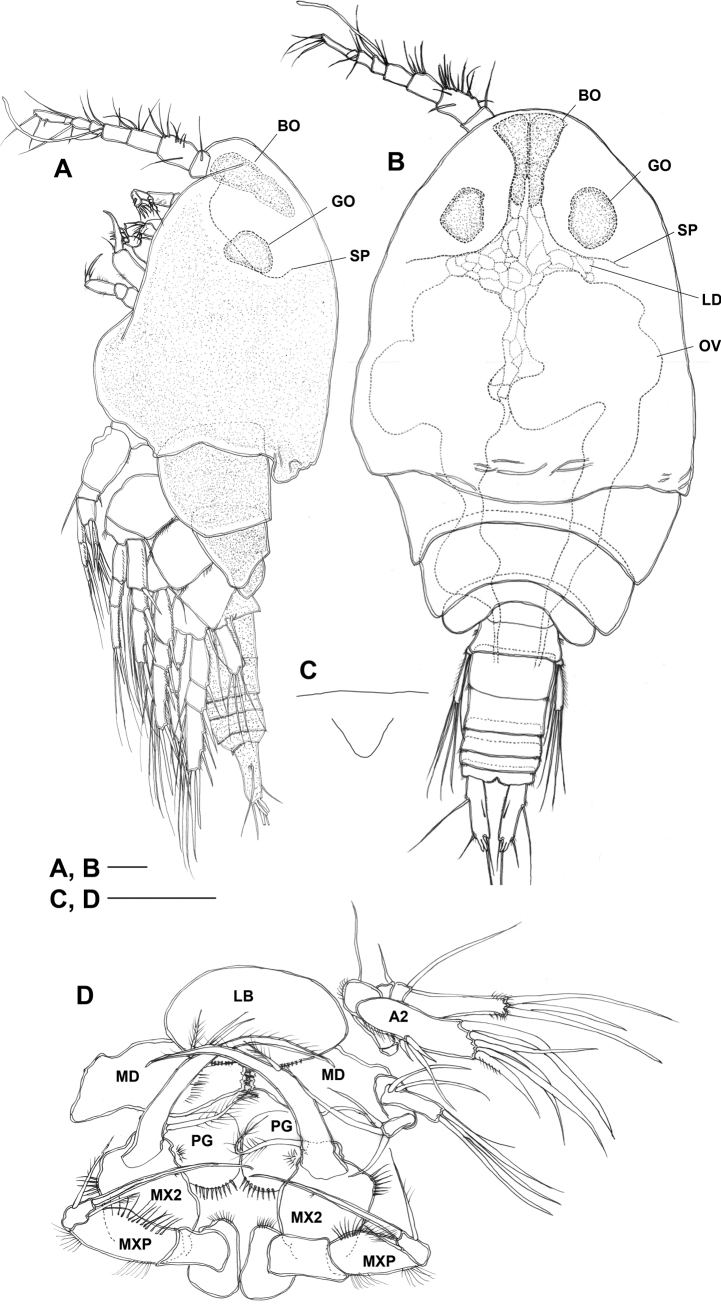
*Gyoromeguttatum* gen. et sp. nov., adult female, holotype **A** habitus, internal structures omitted **B** habitus, dorsal view, surface ornamentation omitted **C** rostrum, ventral view **D** mouthparts, ventral view, right antenna, right mandibular palp, and both maxillules omitted. Abbreviations: A2, antenna; BO, baculiform ocellus; LB, labrum; LD, lipid droplets; MD, mandible; MX2, maxilla; MXP, maxilliped; OV, ovary; PG, paragnath; GO, globular organ; SP, semi-parabolic plate. Scale bars: 0.1 mm.

**Figure 3.**
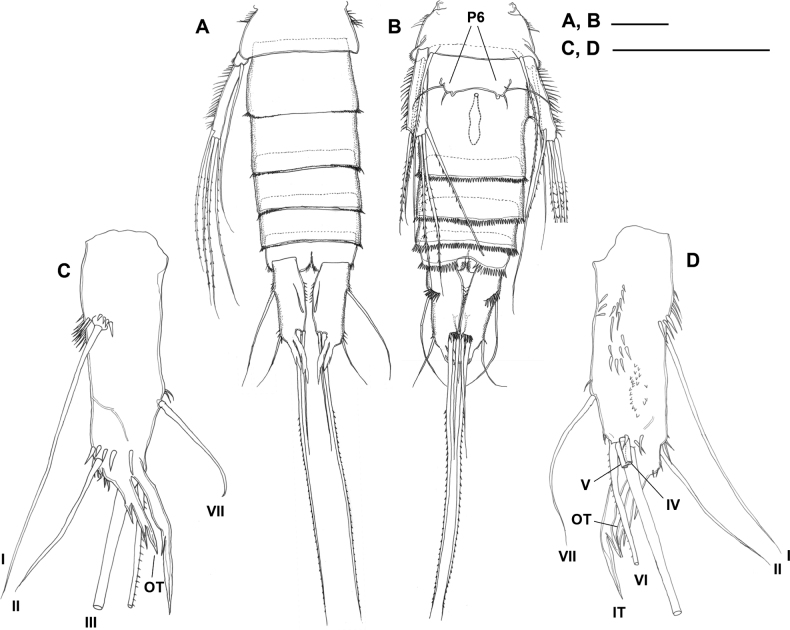
*Gyoromeguttatum* gen. et sp. nov., adult female, paratype **A, B** urosome, dorsal and ventral views, respectively (surface denticles partly omitted to reveal other structures) **C, D** left caudal ramus, dorsal and ventral views, respectively (surface denticles omitted). Abbreviations: P6, sixth pair of legs; I–VII, caudal ramus setae I–VII; IT, inner branching tube-pore; OT, outer branching tube-pore. Scale bars: 0.1 mm.

**Figure 4.**
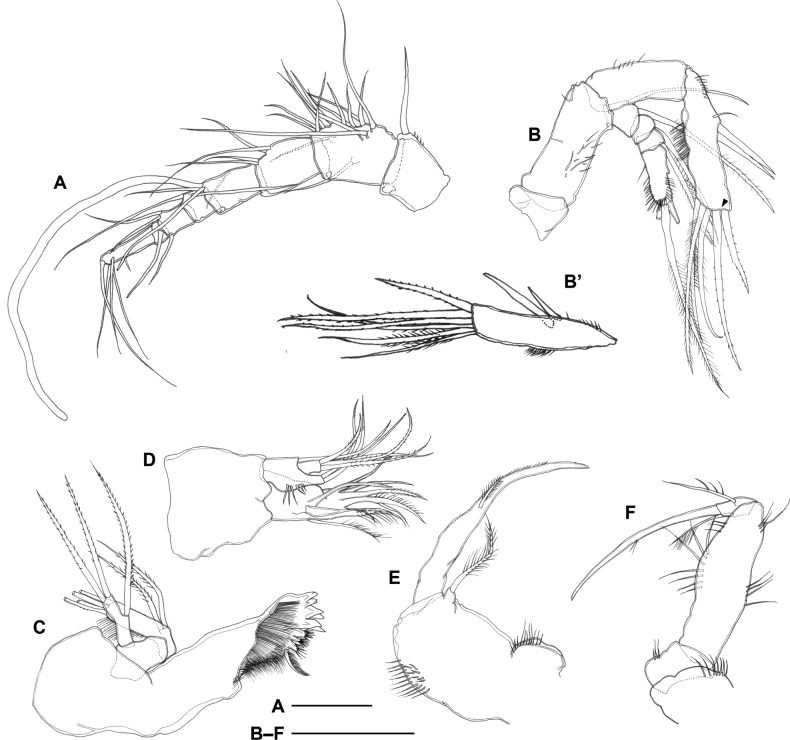
*Gyoromeguttatum* gen. et sp. nov., adult female, paratype **A** right antennule, ventral view **B** right antenna **C** left mandible **D** right maxillule **E** right maxilla **F** left maxilliped, posterior view. Scale bars: 0.1 mm.

**Figure 5.**
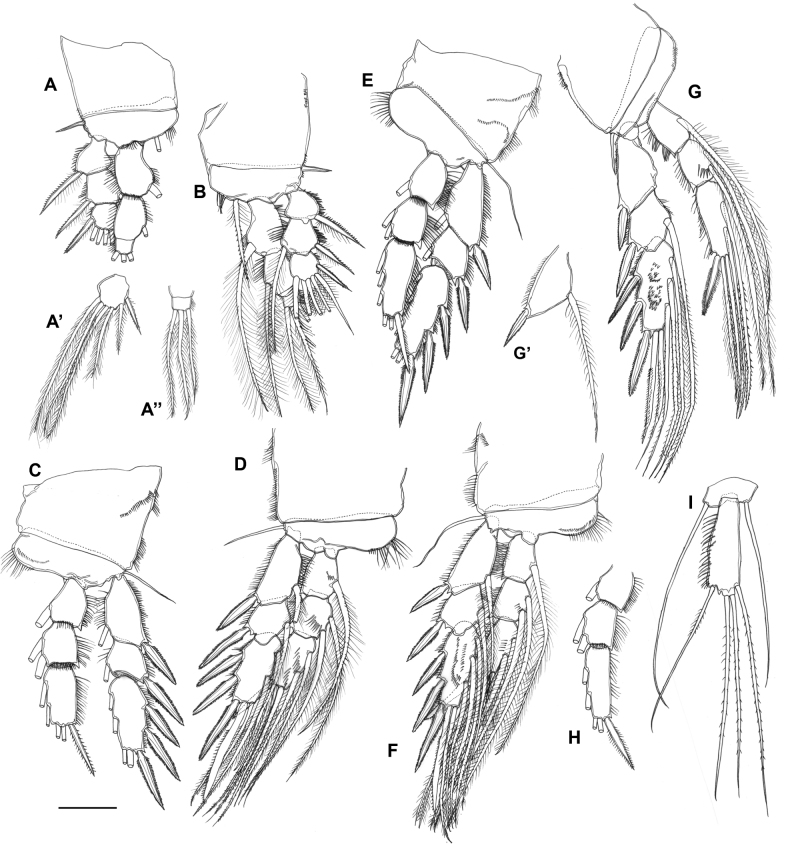
*Gyoromeguttatum* gen. et sp. nov., adult female, paratype **A, B** right leg 1, anterior and posterior views, respectively **A’, A**’’ distal exopodal (A’) and endopodal (A’’) segment of left leg 1, posterior view showing setae at full length **C, D** left leg 2 anterior and posterior views, respectively **E, F** left leg 3, anterior and posterior views, respectively **G** left leg 4, posterior view **H** endopod of left leg 4, anterior view **I** right leg 5, anterior view. Scale bar: 0.1 mm.

**Figure 6.**
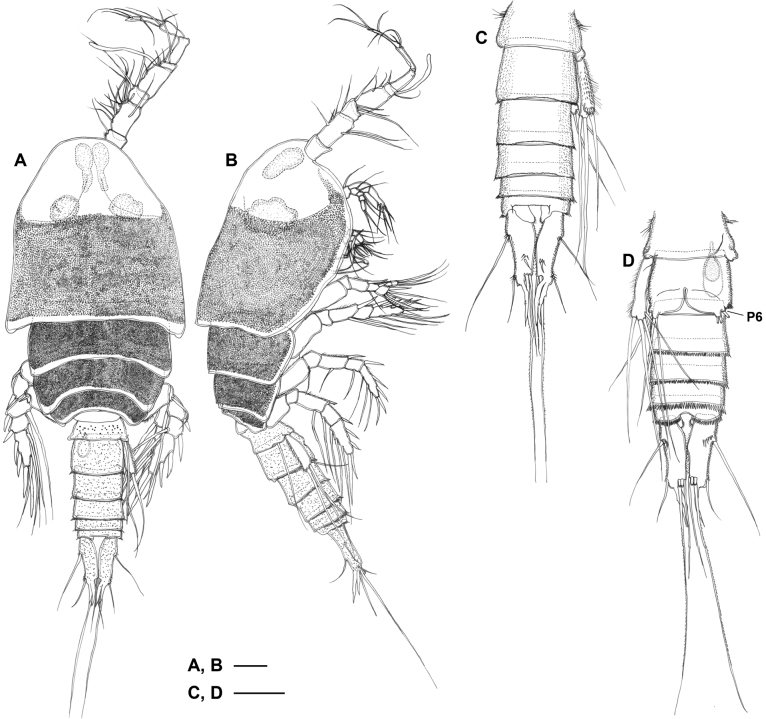
*Gyoromeguttatum* gen. et sp. nov., adult male, paratype **A, B** habitus, internal structures omitted, dorsal and lateral views, respectively **C, D** urosome, dorsal and ventral views, respectively (surface denticles partly omitted to reveal other structures). Abbreviation: P6, leg 6. Scale bars: 0.1 mm.

**Figure 7.**
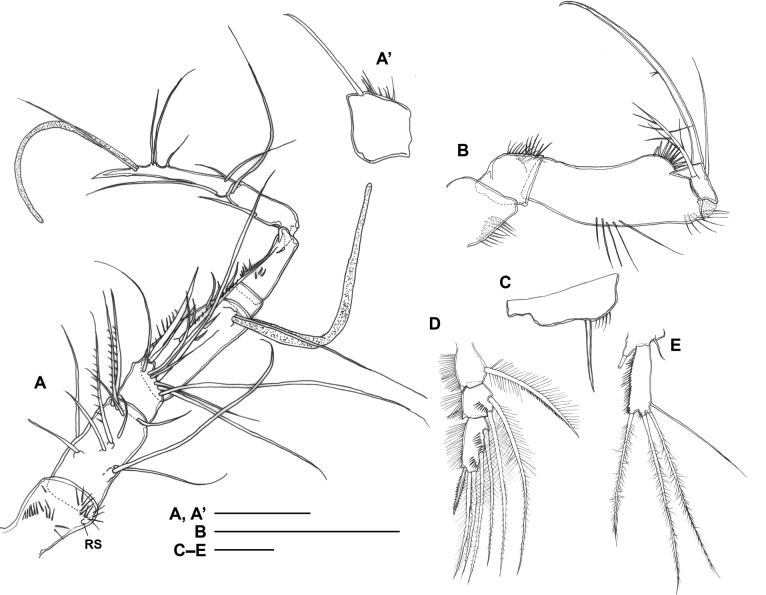
*Gyoromeguttatum* gen. et sp. nov., adult male, paratype **A** left antennule, ventral view **A**’ segment 1 of left antennule showing detached seta **B** right maxilliped, anterior view **C** left basis of leg 1, posterior view **D** endopod of left leg 2, posterior view **E** right leg 5, anterior view. Abbreviation: RS, root of detached seta. Scale bars: 0.1 mm.

Figs 1 , 2 , 3 , 4 , 5 , 6 , 7

##### Type locality.

Japan, Kuroshio region (Off Mie Prefecture, 33°10'N, 136°00'E), epi- or mesopelagic zone (0–935 m depth).

##### Type material.

***Holotype***: Undissected ♀ (1.80 mm) in vial (NSMT-Cr 31562). ***Allotype***: ♂ (1.47 mm), dissected prosome, urosome and appendages mounted on glass slide (NSMT-Cr 31563). ***Paratypes***: One dissected ♀ (1.77 mm) mounted on glass slide (NSMT-Cr 31564), one undissected ♀ (1.64 mm) preserved in 10% neutralized formalin-seawater solution in vial (NSMT-Cr 31565).

##### Description of adult female.

Total body length ranging from 1.64–1.80 mm (*n* = 3). ***Habitus*** (Figs 1, 2A, B) cyclopiform and yellowish. Prosome (Fig. 2A, B) significantly expanded bilaterally, ovoid in dorsal aspect producing vaulted appearance; integument of cephalothorax (except anterior portion) and somites bearing legs 2–4 with dense pattern of minute surface denticles (Fig. 2A). Cephalosome completely fused to first pedigerous somite, forming cephalothorax; ventral surface between maxilliped and leg 1 with distinct protuberance; posterodorsal margin with semicircular lobate extension covering anterior third of leg 2-bearing somite. Anterior part of cephalosome with middorsal pair of baculiform ocelli (BO in Fig. 2A, B) and one pair of globular organs (GO in Fig. 2A, B) each surrounded by thin semi-parabolic plate (SP in Fig. 2A, B) either side of baculiform ocelli; lipid droplets (LD in Fig. 2B) filling up space between and posterior to semi-parabolic plates; ovaries (OV in Fig. 2B) occupying larger part of posterior half of cephalothorax. Rostrum (Fig. 2C) small and triangular, pointing downwards; labrum (LB in Fig. 2D) a rounded lobe with spinules around distal margin; paragnaths (PG in Fig. 2D) represented by semicircular lobes fringed with setulae posteriorly and laterally.

Pedigerous somites bearing legs 2–4 completely separated (Fig. 2A, B); with well-developed pleurotergites, gradually decreasing in width; pleural areas of somites bearing legs 3 and 4 protruding posteriorly.

***Urosome*** (Fig. 3A, B) cylindrical, comprising fifth pedigerous somite, genital double-somite, and three free abdominal somites; all somites with dense pattern of minute surface denticles (Fig. 2A). Leg 5-bearing somite with lateral setular tufts in anterior half. Original segmentation of genital double-somite marked by transverse suture and accompanying spinules dorsally and dorsolaterally; posterior margin with spinules all around; copulatory pore small, located midventrally, immediately posterior to genital slit; copulatory duct well developed, with slight bilateral constriction halfway down its length. Genital double-somite and free abdominal somites with numerous minute tubercles laterally; fourth and fifth urosomites with continuous row of spinules around posterior margin; anal somite with setulae posteromedially and with paired rows of spinules near bases of caudal rami.

***Caudal ramus*** (Fig. 3C, D) ~ 3.3× as long as wide (measured in dorsal aspect); with seven setae, setae I–III and VII slender and naked, setae IV–V broken, represented in all specimens by short basal parts, setae VI slender and pinnate; seta I longer than caudal ramus, originating laterally in proximal third of ramus; seta II arising from outer distal corner; bases of setae IV and V positioned slightly ventral to that of seta VI; seta VI long (Fig. 3A, B), ~ 2.5× length of ramus length; seta VII located dorsally near inner margin at ~ 70% of ramus length; dorsal posterior margin of ramus with two elongate, branching tube-pores (inner one distinctly longer than outer one), covering bases of setae III–VI; ornamentation consisting of spinules on ventral surface of ramus and around bases of setae I–III and, to a lesser extent, VII.

***Antennule*** (Fig. 4A) 8-segmented, ~ 0.25× as long as body length; armature as follows: 1-(1), 2-(15), 3-(4), 4-(3 + ae), 5-(2), 6-(3), 7-(1), 8-(5); all setae naked; segment 1 with inner spinules; aesthetasc on segment 4 well-developed and 0.8× as long as antennule; segments 7 and 8 incompletely fused, original segmentation indicated by transverse surface suture.

***Antenna*** (Fig. 4B, B’) without ornamentation on coxa. Basis unarmed, with setules on posterior surface. Endopod 2-segmented; proximal segment unarmed, with setules along abexopodal margin; distal segment with one minute and two well developed elements laterally and six setae apically, inner margin with short spinules proximally and outer margin with longer spinules in proximal half. Exopod 4-segmented; segments 1–3 with one lateral seta; segment 4 with three apical setae and spinules along inner and outer margins.

***Mandible*** (Fig. 4C). Gnathobase with three well developed bicuspid teeth, three smaller teeth with terminal setular tuft, one hirsute dorsal seta, and transverse row of fine, densely arranged setules. Basis unarmed. Endopod unsegmented, with two proximal setae along inner margin and four distal setae; outer margin with row of setules. Exopod unsegmented, with one inner and two distal setae.

***Maxillule*** (Fig. 4D) 3-segmented, comprising praecoxa, endopod, and compound segment representing fused coxa, basis and exopod. Praecoxal arthrite with two naked setae on anterior surface; medial margin with one plumose seta; distal margin with two naked and three pinnate spines (fused at base to arthrite). Compound segment with few spinules along inner margin; coxa represented by subcylindrical endite with two setae; basal endites with three setae; exopod completely incorporated in segment, represented by single seta. Endopod distinct, with two setae.

***Maxilla*** (Fig. 4E) 2-segmented, comprising syncoxa and allobasis. Syncoxa with setules along outer margin; medial margin with proximal protuberance bearing spinular row; coxal endite represented by small process with one plumose apical seta. Allobasis produced into curved claw with fine pinnules along outer margin and short plumose seta just over halfway claw length.

***Maxilliped*** (Fig. 4F) 3-segmented, comprising syncoxa, basis and endopod. Syncoxa small, articulating with subcylindrical pedestal bearing long spinules at outer distal corner; with few spinules along medial margin. Basis elongate, ~ 3.5× as long as maximum width; unarmed; medial margin slightly expanded, with sparse long spinules in middle third and shorter spinules further distally; outer margin with two groups of long spinules as figured. Endopod small, subrectangular; outer margin with one unipinnate seta; distal margin with long, slender claw accompanied at base by plumose seta; claw with two closely set spinules halfway the inner margin.

***Legs 1–4*** (Fig. 5A–H) with large coxa, narrow basis and 3-segmented rami; without minute surface denticles. Coxa with several spinule rows along outer margin (particularly P2–P4) as figured. Basis with short spine (leg 1) or long naked seta (legs 2–4) on outer margin; inner lobate expansion with numerous long and/or short setules/spinules. Endopod longer (leg 1) or distinctly shorter (legs 2–4) than exopod.

***Leg 1*** (Fig. 5A, B). Basis with long inner spine, extending to middle of enp-2, bipinnate except for plumose proximal quarter; distal margin with anterior spinules near articulation with endopod. Exopodal segments with spinules along outer margins; exp-2 not markedly longer than other segments, with setules along inner margin; outer spines without spinular combs. Endopod indistinctly 3-segmented with transverse surface suture marking original segmentation between enp-2 and -3; outer margins of all segments with spinules, additional spinules along inner margins of enp-1 and -2; enp-1 expanded in distal half forming lobate extension along medial margin; enp-3 small.

***Legs 2–4*** (Fig. 5C–H). Exp-2 markedly shorter than proximal and distal segments. Exopodal spines more robust than in P1. Spinular ornamentation present along outer margins of all exopodal and endopodal segments, and along inner margin of exp-1; few spinules also discernible along inner margin of exp-2. Posterior surface of P3–P4 exp-3, P2 enp-1–3, P3 enp-2 and P4 enp-2–3 with additional spinules. Armature formula as for genus.

***Leg 5*** (Fig. 5I) 2-segmented, comprising baseoendopod and 1-segmented exopod. Baseoendopod apparently fused basally to somite; endopodal lobe obsolete, armature represented by one very long seta (twice length of exopod); outer basal seta very long and naked. Exopod elongate, gradually widening towards distal margin; ~ 2.8× as long as maximum width; with setules along outer margin; armature consisting of one inner, one outer and two terminal setae (all elements sparsely bipinnate); small apical tubercle discernible between outer and outer terminal setae.

***Sixth pair of legs*** (P6 in Fig. 3B) fused medially, forming common plate closing off genital slit; each leg represented by one long outer and one short inner setae. Egg-sac not observed.

##### Description of adult male.

Total body length 1.47 mm (*n* = 1). Sexual dimorphism in prosomal ornamentation, antennule, maxilliped, P2 endopod, P5, P6 and urosomal segmentation.

Prosome resembling that of female except for denticles covering dorsal surface of cephalothorax and pedigerous somites much denser and middorsal protrusion around posterior margin of cephalothorax not expressed (Fig. 6A, B). Urosome (Fig. 6C, D) 6-segmented; denticles covering surface sparser than on prosome; spermatophore located in left half of genital somite; caudal ramus similar to that of female.

***Antennule*** (Fig. 7A, A’) 8-segmented, ~ 0.4× as long as body length; armature as follows: 1-(1), 2-(11), 3-(9), 4-(6 + ae), 5-(1), 6-(0), 7-(2), 8-(7 + ae); segment 1 with spinular pattern on ventral surface; segment 6 with spinules; geniculation between segments 6 and 7; terminal portion of segment 8 digitiform and slowly curved.

***Maxilliped*** (Fig. 7B) 4-segmented; palmar margin of basis with lobate spinular expansion in distal third; medial margin of endopod produced into triangular unguiform projection.

***Legs 1–4*** similar to female condition except for inner basal spine of leg 1 without setulae or spinules (Fig. 7C) and inner seta of proximal endopodal segment of leg 2 (Fig. 7D) displaying pinnate ornamentation along distal half of outer margin (instead of plumose in female).

***Leg 5*** (Fig. 7E) 2-segmented as in female; endopodal seta much shorter, only ~ one-third the length of exopod; exopod ~ 3.1× as long as maximum width; outer margin with spinules.

***Sixth pair of legs*** (P6 in Fig. 6D) symmetrical, each represented by ovoid plate closing off genital aperture and bearing three naked, well developed setae.

##### Etymology.

The specific name is derived from the Latin *guttatum* meaning spotted or speckled and alludes to the dense denticular ornamentation on the male prosome (Fig. 6A, B).

### ﻿Key to planktonic Tisbidae

With the addition of *Gyorome*, four genera in the Tisbidae are now known to inhabit the meso- and bathypelagic oceanic zones. Three of these genera are monotypic while two species were assigned to *Volkmannia* (Boxshall 1979). Bradford and Wells (1983) described both sexes of *Tisbespinulosa* Bradford & Wells, 1983 from a bait trap collected at nearly 600 m below sea level beneath the Ross Ice Shelf in Antarctica. They stated that it belongs among those species of *Tisbe* that display a P1 setation different from the normal type as defined by Volkmann (1979b). Based on aspects of the female antennule, mandible, P1 armature, P5 and genital field the species was regarded as intermediate between *T.finmarchica* (Sars, 1905) and the *T.gracilis*-group. In addition, Bradford and Wells (1983) considered two male characters, the maxilliped and the inner seta of P2 enp-1, that suggested a possible link with the latter group, admitting however that the resemblance in these sexually dimorphic features is not exact. The maxilliped in males of the *T.gracilis*-group displays (a) a lobate spinular expansion along the distal palmar margin of the basis, (b) a terminal endopodal claw which is much shorter than in the female, often slightly sinusoid, and bears a characteristic protuberance (“knee” sensu Volkmann 1979b) along the inner margin, and (c) usually an unguiform projection on the inner margin of the free endopodal segment (this can be absent in some species, e.g., *T.dahmsi* Ivanenko, Ferrari, Defaye, Sarradin & Sarrazin, 2011). The lobate basal expansion and the unguiform endopodal projection are both expressed in the male maxilliped of *T.spinulosa*, however, the endopodal claw is not sexually dimorphic and lacks the proximal protuberance. In all male members of the *T.gracilis*-group the inner seta of the proximal endopodal segment of P2 is transformed into a robust spine, typically displaying a species-specific shape and ornamentation. In *T.spinulosa* this seta is not spiniform but differs from the female condition in its shorter length and more elaborate ornamentation along the proximal outer margin. Although Bradford and Wells (1983) considered including *T.spinulosa* in the *gracilis*-group, they refrained from this course of action due to two characters preventing such an assignment, i.e., the armature pattern on the distal exopodal segment of P1 (3 setae + 3 spines), and the pustulate ornamentation of the body surface. The latter character was viewed as potential supporting evidence for the exclusion of *T.spinulosa* from the genus. Both Gómez et al. (2004) and Ivanenko et al. (2011) cursorily mentioned the species but no new insights emerged from their discussions. Finally, based on the sexual dimorphism of the maxilliped, morphology of P1 and spinular ornamentation of the body, Huys (2021) concluded that *T.spinulosa* must be assigned to *Volkmannia* and formally transferred it to this genus as *V.spinulosa* (Bradford & Wells, 1983). Additional morphological characters in support of its removal from the genus *Tisbe* include the unarmed mandibular basis, the absence of spinular combs on the exopodal spines of P1, the lateral displacement of caudal ramus setae IV–V, and the length:width ratio (> 2.0) of the caudal rami. *Volkmanniaspinulosa* is most closely related to *V.forficula* Boxshall, 1979 and can be differentiated by small differences in the caudal rami and P5 of both sexes. The six species of the *Bathyidia*-lineage can be differentiated by the key below:

**Table:** 

1	P1 exp-2 elongate, markedly longer than exp-1 and exp-2; P1 endopod prehensile, enp-1 and -2 distinctly elongate (at least 2.5–3× as long as maximum width), enp-1 not expanded in distal half, enp-3 minute with outer spine and two terminal setae; P5 ♀ with three endopodal setae *Volkmannia*	**2**
–	P1 exopodal segments subequal in size; P1 endopod non-prehensile, enp-1 and -2 less than twice as long as maximum width, enp-1 with medial lobate expansion in distal half, enp-3 moderately developed or fused to enp-2 forming 2-segmented ramus, with three terminal setae; P5 ♀ with one endopodal seta	**4**
2	P1 endopod ~ 1.7× as long as exopod; P5 ♀ exopod 3× as long as maximum width; caudal ramus ~ 1.8× as long as wide	***V.attenuata***
–	P1 endopod ~ 1.3–1.4× as long as exopod; P5 ♀ exopod 2.5× as long as maximum width; caudal ramus > 2× as long as wide	**3**
3	Caudal ramus 2.2× as long as wide; P5 ♀ outer endopodal seta minute, ~ 1/5 length of exopod, inner seta shorter than exopod; P5 ♂ with two endopodal setae	***V.spinulosa***
–	Caudal ramus 2.65× as long as wide; P5 ♀ outer endopodal seta ~ 1/2 as long as exopod, inner seta longer than exopod; P5 ♂ with one endopodal seta	***V.forficula***
4	Antenna with one seta on exp-1; mandibular endopod with one lateral seta; P1 endopod distinctly 2-segmented	***Neotisbellagigas***
–	Antenna with two setae on exp-1; mandibular endopod with two lateral setae; P1 inner basal spine not sexually dimorphic; P1 endopod distinctly or indistinctly 3-segmented	**5**
5	Cephalosome with paired, frontal, modified eyes; antennary basis unarmed; mandibular endopod with four terminal setae	***Gyoromeguttatum* gen. nov. et sp. nov.**
–	Cephalosome without frontal modified eyes; antennary basis with abexopodal seta; mandibular endopod with five terminal setae	***Bathyidiaremota***

## ﻿Discussion

### ﻿Taxonomic position of *Gyorome* gen. nov. within the Tisbidae

Within the family Tisbidae, *Gyorome* gen. nov. belongs to a close-knit group of exclusively planktonic deepwater genera, including *Bathyidia*, *Neotisbella* and *Volkmannia* (Table 1). Members of this *Bathyidia*-lineage are generally large (in excess of 1 mm) and characterized by the following suite of characters: (a) prosome (cephalothorax and pedigerous somites) with pustulate integument (ornamentation consisting of dense pattern of small tubercles and denticles); (b) female antennule short and compact compared to other free-living tisbid genera, 8-segmented; (c) antenna without seta on proximal endopodal segment; (d) mandibular basis without armature; (e) distal palmar margin of male maxilliped produced into lobate spinular expansion and endopod with unguiform projection; (f) P1–P4 with dense pattern of minute spinules on anterior surface of protopod and rami; (g) P1 spines on middle and distal exopodal segments without spinular combs; (h) inner seta of proximal endopodal segment of P2 modified in male, displaying pinnate ornamentation along distal half of outer margin (instead of plumose in female); and (i) caudal ramus with paired multi-branched tube-pores along posterior margin, displacing setae IV–V towards inner distal corner. At least (a), (c)–(f), (h) and (i) can be considered as shared derived characteristics supporting the monophyly of the four deep water genera. Character states (b) and (g) will require further assessment across the entire family before their potential status as synapomorphies can be ascertained. Within this clade, *Volkmannia* displays the most primitive armature on the female leg 5 with three setae remaining on the endopod while in the other genera the endopodal armature is reduced to a single seta. Similarly, the male P5 in *V.spinulosa* (but not in *V.forficula*, unknown in *V.attenuata*) exhibits two endopodal setae vs only one seta in the remaining genera. Members of *Volkmannia* also (a) have the most primitive armature pattern on the mandibular endopod, displaying three lateral and six terminal setae (vs at most two lateral and five apical setae in the other genera), (b) display elongation of the middle segment of the P1 exopod (vs all exopodal segments subequal), (c) possess a prehensile P1 endopod with distinctly elongate enp-1 and -2, and a minute apical segment (enp-3) bearing one outer spine and two terminal setae (vs non-prehensile without distinct elongation of segments and three setae on terminal segment), and (d) show three well developed setae on the female sixth legs. *Bathyidia*, *Neotisbella*, and *Gyorome* collectively form the sister group of *Volkmannia* and, in addition to the setal reductions in the mandibular endopod and P5 baseoendopod mentioned above, share the characteristic shape of the proximal segment of P1 endopod. Unlike in other tisbid genera this segment has undergone allometric growth medially, forming a distinct lobate expansion in its distal half from where the inner seta originates.

**Table 1.** Morphological comparison of pelagic genera in the family Tisbidae.

	*Volkmannia*	*Bathyidia*	*Gyorome* gen. nov.	*Neotisbella*
Enlarged modified eyes	absent	absent	present	absent
Antennary basis	with seta	with seta	unarmed	unarmed
Antennary exopod armature	2-1-1-3	2-1-1-3	2-1-1-3	1-0-1-3
Mandibular endopod armature	3 lateral + 6 terminal setae	2 lateral + 5 terminal setae	2 lateral + 4 terminal setae	1 lateral + 4 terminal setae
P1 inner basal spine ♂	as in ♀	as in ♀	sexually dimorphic	sexually dimorphic
P1 exp-2	elongate, longer than exp-1	as long as exp-1	as long as exp-1	as long as exp-1
P1 endopod segmentation	3-segmented	3-segmented	indistinctly 3-segmented	2-segmented
P1 endopod	prehensile; enp-1 and -2 distinctly elongate (at least 2.5× as long as maximum width), enp-3 minute	non-prehensile; enp-1 and -2 less than twice as long as maximum width, enp-3 moderately developed	non-prehensile; enp-1 and -2 less than twice as long as maximum width, enp-3 moderately developed	non-prehensile; enp-1 and compound enp-2 subequal, ~ 2× as long as maximum width
P1 enp-1 shape	not expanded distally	expanded in distal half	expanded in distal half	expanded in distal half
P1 distal endopodal segment armature	outer spine + 2 terminal setae (on enp-3)	3 terminal setae (on enp-3)	3 terminal setae (on enp-3)	1 lateral and 3 terminal setae (on enp-2)
P5 exopod ♀/♂ armature	4 long + 1 short setae	4 long + 1 vestigial setae	4 long setae	4 long + 1 short setae
P5 endopod ♀ armature	3 setae	1 seta	1 seta	1 seta
P5 endopod ♂ armature	1–2 setae	1 seta	1 seta	1 seta
P6 ♀ armature	3 well developed setae	2 minute setae	1 minute and 1 well developed setae	2 minute and 1 well developed setae

*Gyorome* appears most closely related to *Neotisbella* based on the unarmed antennary basis (loss of abexopodal seta), further reduction in mandibular armature (endopod with only four terminal setae instead of 5–6), and the virtually identical morphology of the P1 endopod (indistinctly 3-segmented in *Gyorome*, with original segmentation marked by transverse surface suture between enp-2 and -3; genuinely 2-segmented in *Neotisbella*). Both genera also share, to a certain degree, a bilaterally and dorsoventrally expanded prosome, giving it a vaulted appearance. *Neotisbella* differs from *Gyorome* in the reduced armature of the antennary exopod (1-0-1-3 vs the ancestral pattern 2-1-1-3 retained *Gyorome*), the presence of only one lateral seta (vs two) on the mandibular endopod, the sexual dimorphism expressed in the inner basal spine of leg 1 (transformed into a seta in the male), and short caudal ramus setae IV and V. The new genus can readily be differentiated from *Neotisbella* by the presence of paired, frontal, modified eyes, and the reduction in the number of armature elements on the P5 exopod in both sexes.

### ﻿Caudal ramus morphology

**Figure 8.**
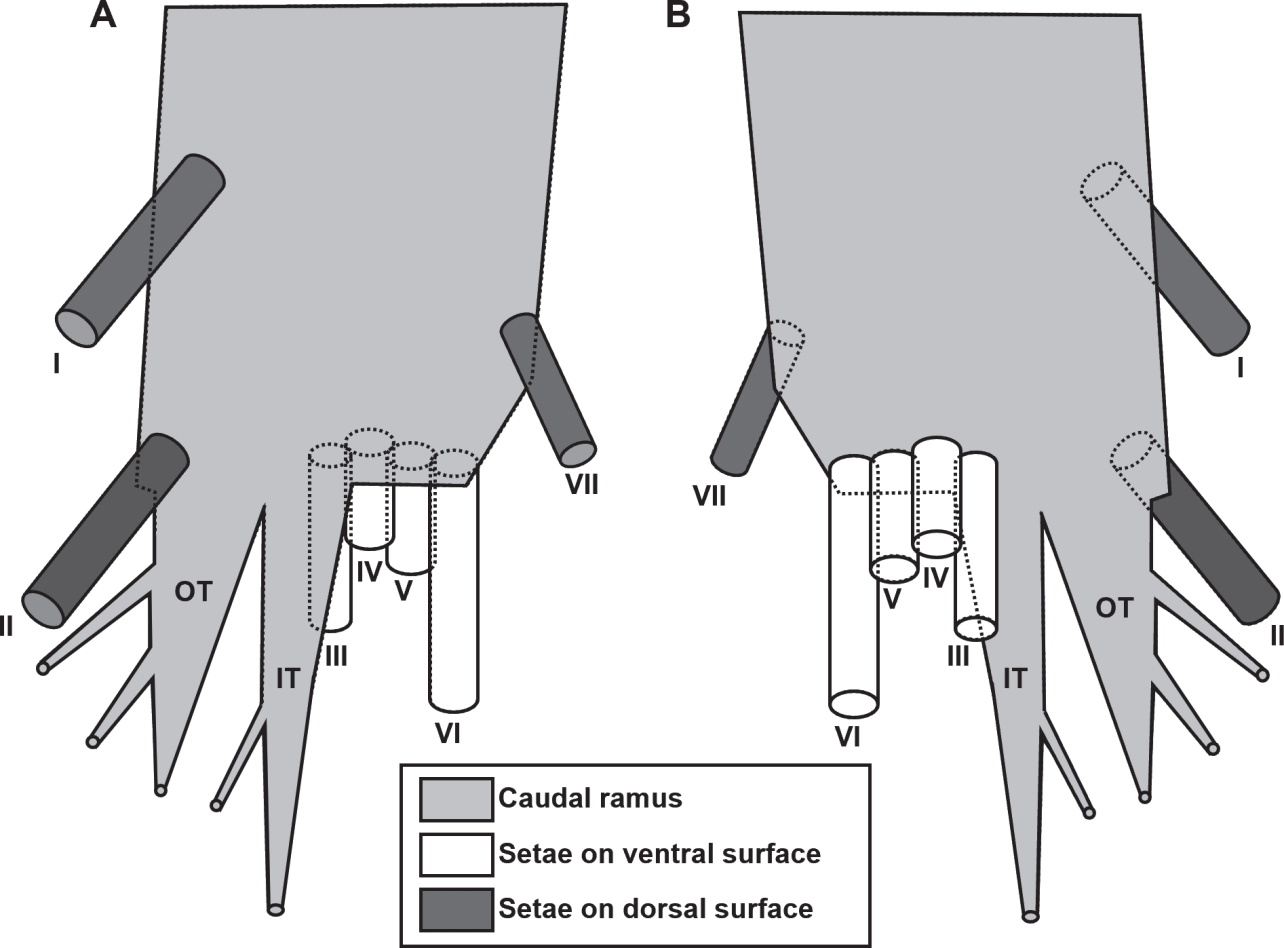
Schematic diagram of left caudal ramus of *Gyorome* gen. nov. **A** dorsal view **B** ventral view. I–VII, caudal ramus setae I–VII; IT, inner branching tube-pore; OT, outer branching tube-pore.

Huys (1988) proposed a standard terminology for the seven caudal setae displayed by the generalized paramesochrid caudal ramus and stated that it is universally applicable to all harpacticoid families. The system was subsequently adopted by Huys and Boxshall (1991) who extended its application to all copepod orders and posited that the hypothetical copepod ancestor exhibited no more than seven setae on the caudal ramus. It had previously been pointed out that the report by Lang (1948) of eight setae in *Canuella* Scott & Scott, 1893 and *Sunaristes* Hesse, 1867 (Canuellidae now removed from the Harpacticoida) was based on observational errors (Huys 1988). Similarly, Boxshall (1979) reported up to nine elements on the caudal rami of three meso/bathypelagic genera (*Bathyidia*, *Neotisbella*, *Volkmannia*) in the family Tisbidae. The supernumerary elements in these genera originate from the posterior margin of the caudal ramus between setae III and IV and are typically thin-walled and flaccid. Huys and Boxshall (1991: fig. 3.12.1B, C) re-examined *Bathyidiaremota* Farran, 1926 and revealed that the additional “setae” were extremely elongate and complex multi-branching tube-pores rather than articulating armature elements (Fig. 8: IT, OT). Examination of the types of *Neotisbellagigas* Boxshall, 1979 (NHMUK reg. nos 1977.266–232), *Volkmanniaforficula* (NHMUK reg. nos 1977.233–241), and *V.attenuata* Boxshall, 1979 (NHMUK reg. no. 1977.324) confirmed the presence of similar caudal ramus tube-pores while inspection of material of other tisbid genera failed to reveal such structures. The discovery of these pores in *Gyorome* (Fig. 3A, C, D: IT, OT) points to a common ancestry of the deepwater planktonic genera in the Tisbidae. The function of these tube-pores is as yet unknown but their complex morphology, in conjunction with the significant displacement of setae IV and V towards the inner distal corner of the ramus, is here regarded as a synapomorphy supporting the monophyly of the four pelagic genera in the family. The only exception in this lineage is *Volkmanniaspinulosa* (Bradford & Wells, 1983) which apparently lacks such tube-pores, however, the distinct gap between seta III and the laterally displaced setae IV and V suggests that these transparent structures were overlooked in the original description (Bradford and Wells 1983: fig. 7d, h, i).

### ﻿Modified naupliar eyes in Harpacticoida

Adult copepods typically have tripartite naupliar eyes consisting of three fused ocellar units (paired dorsolateral ocelli and one unpaired ventral ocellus). Each unit is made up of a retinal photoreceptor sphere, a tapetal layer and a surrounding pigment cup. However, the evolution of different designs from this simple eye generated more novelty and diversity in form than that of the more complex compound eye types found across the rest of the Crustacea (Steck et al. 2023). In several lineages secondary modifications of the ocellar components of the typical naupliar eye have evolved, ranging from complete loss to extreme enlargement, separation of the cups into three independent eyes, and the addition of structures used to focus light onto the retina such as crystalline or cuticular lenses. Extreme eye modification has been histologically documented in at least four orders, including the Calanoida (e.g., Pontellidae, *Cephalophanes*), Cyclopoida (e.g., Corycaeidae, Sapphirinidae), Siphonostomatoida (Caligidae) and Harpacticoida (e.g., Elofsson 1966; Huys and Böttger-Schnack 1994; Land 1984, 1988; Nishida et al. 2002; Vaissière 1961). While most species in the latter order are thought to have typical naupliar eyes or have secondarily lost them, there are a few notable exceptions. Within the Harpacticoida elaborate eyes are only found in members of the planktonic subfamily Miraciinae (Miraciidae) with three of its four monotypic genera (*Miracia* Dana, 1846; *Oculosetella* Dahl, 1895; *Distioculus* Huys & Böttger-Schnack, 1994) displaying eyes of the telescopic type with double lenses oriented in the same light path with one distal to the other. The paired ocelli of the large anteriorly directed naupliar eyes each have an exterior lens of unknown origin, with both lenses being linearly arranged along the frontal margin of the cephalic shield, as well as a second lens directly in front of the retina (Claus 1891; Elofsson 1966; Huys and Böttger-Schnack 1994). Unlike the Corycaeidae and Sapphirinidae which also exhibit telescopic eyes, the dorsolateral ocelli have not undergone lateral displacement in the three miraciinid genera and the basic tripartite structure of the naupliar eye is retained. Phylogenetic analysis suggests that the frontal lenses were secondarily lost in the fourth genus, *Macrostella* A. Scott, 1909, possibly because of its intimate association with filamentous cyanobacteria (Huys and Böttger-Schnack 1994).

Some members of the genus *Paradactylopodia* Lang, 1944 (Dactylopusiidae) display lens-like structures on the frontal part of the cephalothorax. In *P.spinipes* (Brady, 1910) and *P.oculata* (Gurney, 1927) paired subintegumental lenses are positioned near the bases of the antennules (Brady 1910; Gurney 1927) and discernible in both dorsal and lateral aspects. In *P.trioculata* Hicks, 1988a the frontal portion of the cephalothorax displays lens-like structures set in a triangle with the anteriormost located at the base of the rostrum (note that it is conceivable that the latter was overlooked in *P.spinipes* and *P.oculata*). According to Hicks (1988a) the structures in *P.trioculata* are not merely sacs containing deposits of oil but genuine corneal lenses with high refractive properties. Two species in the speciose family Laophontidae, *Heterolaophonteoculata* (Gurney, 1927) and *Laophontepseudoculata* Krishnaswamy, 1959, also display paired refringent lens-like structures, but nothing is known about their visual function. Interestingly, both *P.trioculata* and *L.pseudoculata* were collected from wood infested with shipworms, the former from a waterlogged teredinid bored log at 51 m depth, the latter from floating logs.

The photoreceptors displayed in *Gyoromeguttatum* are of a level of complexity not previously observed in the Tisbidae. The majority of free-living tisbids display simple tripartite naupliar eyes such as in members of the genera *Drescheriella* (Dahms and Bergmans 1988), *Paraidya* (Humes and Ho 1969), *Sacodiscus* (Sars 1904, 1905), *Scutellidium* (Branch 1974; Itô 1976), *Tisbe* (Bergmans 1979; Bocquet 1951; Vaissière 1961; Volkmann 1979b; Chullasorn et al. 2009), *Tisbella* (Volkmann 1979a), and *Tisbintra* (Ummerkutty 1961). No photoreceptors have been reported in the deepwater genera *Bathyidia*, *Neotisbella*, and *Volkmannia* (Boxshall 1979), however, it remains unclear whether this absence is genuine, or the coloration had already disappeared in the preserved material. The paired enlarged modified eyes in *G.guttatum* essentially each consist of three major components, i.e., (a) a baculiform ocellus (Fig. 2A, B: BO), (b) a semi-parabolic plate (Fig. 2A, B: SP) and (c) a globular organ (Fig. 2A, B: GO). The position and close connection between the large rod-shaped ocelli (Fig. 2A, B: BO) suggest that they are homologous with the paired dorsolateral ocelli expressed in the basic tripartite naupliar eye of most copepods. The unpaired ventral ocellus was not observed in the present study and is probably very reduced or absent.

The semi-parabolic plates bear a superficial resemblance to the semi-parabolic reflective mirrors that replace the tapetal and pigment cells in the paired eyes in members of the calanoid genus *Cephalophanes* Sars, 1907 (Phaennidae) (Nishida et al. 2002) and ostracods belonging to the genus *Gigantocypris* Müller, 1895 (Cypridinidae) (Land 1978, 1984; Nilson 1997). However, the reflectors in these taxa are distinctly colored and have a multilayer structure made up of stacks of thin platelets of putative chitinous material while in *G.guttatum* the semi-parabolic plates are thin and colorless, casting doubt on their reflective potential. Based on muscle anatomy, Nishida et al. (2002) suggested that *Cephalophanes* species can control reflector direction, making their eyes one of the most effective broadband light detectors in the invertebrates; no such musculature was observed in association with the semi-parabolic plates of *G.guttatum*. It is postulated here that these plates merely serve as partitions, separating the spaces occupied by the ocelli and globular organs from the lipid droplets (LD in Fig. 2B), ovary (OV in Fig. 2B) and other organs. This space delimitation and compartmentalization, in conjunction with the apparent absence of dense surface ornamentation in the anterior portion of the cephalothorax, conceivably reduces or minimizes potential interference with the amount of incident light reaching the retinal cells in the ocellar region. With only two receptor cells in each reflector focal area, it is unlikely that the eyes of *Cephalophanesrefulgens* Sars, 1907 have any image-resolving power; however, the presence of parabolic mirrors that direct light back to the retinal cells from all frontal angles optimizes their light-gathering efficiency in deep-sea habitats. It has been suggested that these large eyes are likely to aid in foraging in low light conditions and gut contents analysis of *Cephalophanes* spp. revealed that these detritivores feed primarily on the shower of carcasses (“Leichenregen”) falling from the upper layers of the water column (Nishida et al. 2002; Steuer 1928). The visual detection of these carcasses is potentially facilitated by luminous bacteria that are commonly found associated with them and as such act as biomarkers of detrital food (Ohtsuka et al. 2019). It is unlikely that the presence of enlarged ocelli in *G.guttatum*, which coincidentally occurs in the same habitat as *Cephalophanes* spp., is related to either mate recognition or predator avoidance. Although no information exists on its overall photosensitivity and light-mediated behaviors, we speculate that food detection in *G.guttatum* is also directly mediated by vision. The development of specialized eyes in this species can be interpreted as the product of convergent evolution that, as in *Cephalophanes*, may provide a means for detecting bioluminescent food particles in oligotrophic mesopelagic environments.

The paired globular organs in *G.guttatum* (Fig. 2A, B: GO) are reminiscent of the paired “accessory photoreceptors” observed in some species of *Calanus* Leach, 1816 (Frost 1974). These receptors, collectively called Gicklhorn’s organ, are supplied by a pair of nerves arising laterally from the central nervous system, independent of the optic nerves. Although their innervation was not investigated, the location of the globular organs in *G.guttatum* suggests that they are homologous with the paired Gicklhorn’s organ documented in various calanoids, cyclopoids and harpacticoids (Dudley 1972; Elofsson 1966, 1970, 1971; Frost 1974; Gicklhorn 1930). The organ has variously been interpreted as a non-visual light-sensing structure, an internal chemosensor or a structure involved in controlling the release of neurosecretory products (Elofsson 1966, 1970, 1971) while recent studies using antibody neural tracing suggested that the paired receptors of the Gicklhorn’s organ may be homologous to the arthropod compound eye (Frase and Richter 2020). Pending the arrival of convincing behavioral or physiological evidence, the function and evolutionary origin of this organ remain enigmatic as ever (Steck et al. 2023).

### ﻿Colonization of the open pelagic

Within the Tisbidae only members of the four genera of the *Bathyidia*-lineage are strictly holoplanktonic and oceanic. They inhabit the mesopelagic and bathypelagic zones but are only rarely encountered in plankton samples. *Bathyidiaremota* is typically bathypelagic and has only been found on three occasions in the North Atlantic Ocean since its original description nearly one century ago. Farran (1926) discovered the female holotype in the Bay of Biscay in a plankton haul taken between 1,370 and 1,830 m depth. Deevey and Brooks (1977) subsequently recorded the male at 1,000–1,500 m in the Sargasso Sea while Boxshall (1979) reported both sexes off the Cape Verde Islands at 1,000–1,250 m depth. The species is further only known from a single outlier in the Arabian Sea where Böttger-Schnack (1996) recorded it at 1,050–1,850 m together with a second, as yet undescribed, species of *Bathyidia*. *Neotisbellagigas* has not been recorded again since its original description from mesopelagic depths (300–900 m) in the northeastern Atlantic (Boxshall 1979). The exact depth at which *Gyoromeguttatum* was collected is unknown (0–935 m) but it appears that it assumes a mesopelagic depth distribution. Members of the genus *Volkmannia* are found at both mesopelagic and bathypelagic depths. The type species, *V.forficula*, is known from a single plankton haul taken between 410 and 890 m depth off the Cape Verde Islands (Boxshall 1979). *Volkmanniaattenuata* is a typical bathypelagic species with records from the northeastern Atlantic (3,760–3,920 m) (Boxshall 1979) and the Clarion-Clipperton Zone in the Eastern Pacific (4,123 m) (Khodami et al. 2017). Finally, *V.spinulosa* was obtained from a bait bottle containing seal and fish meat which had been deployed near the sea floor beneath the Ross Ice Shelf (Antarctica) where the sea floor is 597 m below sea level and the water column 237 m thick (Bradford and Wells 1983). Gut contents analysis revealed that *V.spinulosa* had been feeding on the bait but it remains unknown whether this necrophagous (scavenging) habit is the only feeding strategy of the species, or indeed can be extrapolated to other members of the *Bathyidia*-lineage. Some species of the family Tisbidae are known to be omnivores and opportunistic feeders (Hicks and Coull 1983) and scavenging behaviour has previously been observed in *Tisbefurcata* (Baird, 1837) (Garstang 1900) and other members of the genus (Lee 2004; Lee and Morton 2004). Although the feeding strategy of *Gyoromeguttatum* is not revealed, its large, vaulted prosome suggests an opportunistic macrophage that has adopted gorging. The flexible integument and posterodorsal extension of the prosome (in females only) presumable allows for considerable lateral and dorsal distension of the midgut in the similar way to the misophrioid one reported by Boxshall and Roe (1980).

Occasionally, other tisbid species have been recorded from the plankton in the neritic zone, but in most cases, these are temporarily displaced littoral forms (Wells 1970). Similarly, some littoral Tisbidae are known to disperse by clinging to marine algae (e.g., *Sargassum*) drifting in the open ocean currents but such species are not permanent members of the plankton and should be regarded as expatriated forms (Yeatman 1962). In seagrass beds, some tisbids as well as many other phytal harpacticoids demonstrate active emergence, particularly during nighttime, and their entry into the column appears to be linked to precopulatory mate behavior, as evidenced by the predominance of adult males and copepodid V females (Bell et al. 1988; Hicks 1988b; Walters and Bell 1986).

## Supplementary Material

XML Treatment for Gyorome

XML Treatment for Gyorome guttatum

## ﻿Additional information

### Author ORCIDs

Sota Komeda https://orcid.org/0009-0009-4564-1921

Susumu Ohtsuka https://orcid.org/0000-0001-6018-7442

Rony Huys https://orcid.org/0000-0003-2411-7003

### Data availability

All of the data that support the findings of this study are available in the main text.

## References

[B1] Anonymous (2010) Opinion 2257 (Case 3467). Zosimidae Seifried, 2003 (Crustacea, Copepoda, Harpacticoida): Emendation of spelling to Zosimeidae to remove homonymy with Zosiminae Alcock, 1898 (Crustacea, Decapoda, Xanthidae). Bulletin of Zoological Nomenclature 67(4): 334–335. 10.21805/bzn.v67i4.a2

[B2] Avdeev GV (1982) Novye vidy garpaktitsidnykh kopepod–Parasitov os’minigov severo-zapadnoí chasti Tikhogo okeana. New species of harpacticoid copepods, parasites of octopuses in the north-western Pacific. Parazitologiia 16: 107–116. [In Russian with English summary]

[B3] Avdeev GV (1983) Novye garpaktikoidnye kopepody semeĭstva Tisbidae—Parasity os’minogov v more Rossa. New harpacticoid copepods (Tisbidae), parasites of octopuses in the Ross Sea. Zoologicheskij Zhurnal 62: 1775–1785. [In Russian with English summary]

[B4] Avdeev GV (1986) New harpacticoid copepods associated with Pacific cephalopods. Crustaceana 51(1): 49–65. 10.1163/156854086X00052

[B5] Avdeev GV (2010) *Amplipedicolapectinatus* gen. et sp. n. (Copepoda, Harpacticoida, Tisbidae), a parasite of octopuses in the Bering Sea. Crustaceana 83(11): 1363–1370. 10.1163/001121610X535663

[B6] Baird W (1837) The natural history of the British Entomostraca. Magazine of Zoology and Botany 1(1837): 35–41. [309–333, 514–526, plates VIII–X, XV.] [continued without copepod records in 2 (1838), 132–144, 400–412, plate V]

[B7] Becker K-H (1974) Eidonomie und Taxonomie abyssaler Harpacticoidea (Crustacea, Copepoda). Teil 1. Cerviniidae – Ameiridae. Meteor Forschungs-Ergebnisse 18(D): 1–28.

[B8] Bell SS Hicks GRF Walters K (1988) Active swimming in meiobenthic copepods of seagrass beds: Geographic comparisons of abundance and reproductive characteristics. Marine Biology 98(3): 351–358. 10.1007/BF00391111

[B9] Bergmans M (1979) Taxonomic notes on species of *Tisbe* (Copepoda, Harpacticoida) from a Belgian Sluice dock. Zoologica Scripta 8(1–4): 211–220. 10.1111/j.1463-6409.1979.tb00633.x

[B10] Bocquet C (1951) Recherches sur *Tisbe* (= *Idyaea*) *reticulata*, n. sp. Essai d’analyse génétique du polychromatisme d’un Copépode Harpacticoïde. Archives de Zoologie Expérimentale et Générale 87: 335–416.

[B11] Boeck A (1873) Nye Slægter og Arter af Saltvands-Copepoder. Forhandlinger i Videnskabsselskabet i Kristiania 1872: 35–60.

[B12] Böttger-Schnack R (1996) Vertical structure of small metazoan plankton, especially non-calanoid copepods. I. Deep Arabian Sea. Journal of Plankton Research 18(7): 1073–1101. 10.1093/plankt/18.7.1073

[B13] Boxshall GA (1979) The planktonic copepods of the northeastern Atlantic Ocean: Harpacticoida, Siphonostomatoida and Mormonilloida. Bulletin of the British Museum (Natural History). Bulletin of the British Museum, Natural History. Zoology 35: 201–264. 10.5962/p.20454

[B14] Boxshall GA Roe HSJ (1980) The life history and ecology of the aberrant bathypelagic genus *Benthomisophria* Sars, 1909 (Copepoda: Misophrioida). Bulletin of the British Museum (Natural History). Zoology: Analysis of Complex Systems, ZACS 38: 9–41.

[B15] Bradford JM Wells JBJ (1983) New calanoid and harpacticoid copepods from beneath the Ross Ice Shelf, Antarctica. Polar Biology 2(1): 1–15. 10.1007/BF00258279

[B16] Brady GS (1910) Die marinen Copepoden der Deutschen Südpolar-Expedition 1901–1903. I. Über die Copepoden der Stämme Harpacticoida, Cyclopoida, Notodelphyoida und Caligoida. Deutsche Südpolar-Expedition 11 (= Zoologie 3): 497–594. [Plates LII–LXIII.]

[B17] Branch GM (1974) *Scutellidiumpatellarum* n. sp., a harpacticoid copepod associated with *Patella* spp. in South Africa, and a description of its larval development. Crustaceana 26(2): 179–200. 10.1163/156854074X00550

[B18] Bresciani J Lützen J (1994) Morphology and anatomy of *Avdeeviaantarctica*, new genus, new species (Copepoda: Harpacticoida: Tisbidae), parasitic on an antarctic cephalopod. Journal of Crustacean Biology 14(4): 744–751. 10.2307/1548868

[B19] Bröhldick KST (2005) Results of the DIVA-1 expedition of RV “Meteor” (Cruise M48/1). A new taxon of Idyanthidae (Copepoda, Harpacticoida) from the deep sea of the Angola Basin. Organisms, Diversity & Evolution 5(Supplement 1): 43–57. 10.1016/j.ode.2004.10.004

[B20] Bruce JR Colman JS Jones NS [Eds] (1963) Marine fauna of the Isle of Man. Liverpool Marine Biological Committee Memoirs on typical British marine Plants and Animals 36: 1–307.

[B21] Chullasorn S Dahms H-U Schizas NV Kangtia P (2009) Phylogenetic inferences of *Tisbe* Lilljeborg, 1853 (Copepoda, Harpacticoida) with *Tisbethailandensis* sp. nov. from Thailand. Hydrobiologia 627(1): 1–17. 10.1007/s10750-009-9711-2

[B22] Claus C (1866) Die Copepoden-Fauna von Nizza. Ein Beitrag zur Charakteristik der Formen und deren Abänderungen “im Sinne Darwin’s”. Schriften der Gesellschaft zur Beförderung der gesammten Naturwissenschaften zu Marburg 1(Supplement): 1–34, plates I–V.

[B23] Claus C (1891) Ueber die Gattung *Miracia* Dana mit besonderer Berücksichtigung ihres Augen-Baues. Arbeiten aus dem Zoologischen Instituten der Universität Wien 9: 267–284.

[B24] Coull BC (1970) Shallow water meiobenthos of the Bermuda platform. Oecologia 4(4): 325–357. 10.1007/BF00393393 28309716

[B25] Coull BC Herman SS (1970) Zoogeography and parallel level-bottom communities of the meiobenthic Harpacticoida (Crustacea, Copepoda) of Bermuda. Oecologia 5(4): 392–399. 10.1007/BF00815503 28309790

[B26] Cremonte F Puebla C Tillería J Videla V (2015) Histopathological survey of the mussel *Mytiluschilensis* (Mytilidae) and the clam *Garisolida* (Psammobiidae) from southern Chile. Latin American Journal of Aquatic Research 43(1): 248–254. 10.3856/vol43-issue1-fulltext-21

[B27] Dahl F (1895) Die Schwarmbildung pelagischer Thiere. Zoologischer Anzeiger 18: 168–172.

[B28] Dahms H-U Bergmans M (1988) Postembryonic development of *Tisbegracilis* (T. Scott) (Copepoda, Harpacticoida). Zoologica Scripta 17(4): 357–369. 10.1111/j.1463-6409.1988.tb00112.x

[B29] Dahms H-U Dieckmann GS (1987) *Drescheriellaglacialis* gen. nov., sp. nov. (Copepoda, Harpacticoida) from Antarctic sea ice. Polar Biology 7(6): 329–337. 10.1007/BF00293223

[B30] Dahms H-U Schminke HK (1992) Sea ice inhabiting Harpacticoida (Crustacea, Copepoda) of the Weddell Sea (Antarctica). Bulletin de l’Institut royal des Sciences naturelles de Belgique. Biologie 62: 91–123.

[B31] Dahms H-U Bergmans M Schminke HK (1990) Distribution and adaptations of sea ice inhabiting Harpacticoida (Crustacea, Copepoda) of the Weddell Sea (Antarctica). Pubblicazione della Stazione Zoologica di Napoli, I. Marine Ecology 11(3): 207–226. 10.1111/j.1439-0485.1990.tb00240.x

[B32] Dana JD (1846) Notice of some genera of Cyclopacea. The American Journal of Science and Arts 1(2): 225–230.

[B33] Deevey GB Brooks AL (1977) Copepods of the Sargasso Sea off Bermuda: Species composition, and vertical and seasonal distribution between the surface and 2000 m. Bulletin of Marine Science 27: 256–291.

[B34] DeWitt HH (1962) A new genus and species of zoarcid fish from McMurdo Sound, Antarctica. Copeia 1962(4): 819–826. 10.2307/1440684

[B35] Dinet A (1974) Espèces nouvelles de Copépodes Harpacticoïdes (Crustacea) des sédiments profonds de la dorsale de Walvis. Archives de Zoologie Expérimentale et Générale 115: 549–577.

[B36] Dudley PL (1972) The fine structure of a cephalic sensory receptor in the copepod *Doropygusseclusus* Illg (Crustacea: Copepoda: Notodelphyidae). Journal of Morphology 138(4): 407–431. 10.1002/jmor.1051380403 30366485

[B37] Dvoretsky AG Dvoretsky VG (2013) Copepods associated with the red king crab *Paralithodescamtschaticus* (Tilesius, 1815) in the Barents Sea. Zoological Studies 52(1): 1–17. 10.1186/1810-522X-52-17

[B38] Dvoretsky AG Dvoretsky VG (2023) Epibionts of an introduced king crab in the Barents Sea: A second five-year study. Diversity 15(1): 1–29. 10.3390/d15010029

[B39] Elofsson R (1966) The nauplius eye and frontal organs of the non-Malacostraca (Crustacea). Sarsia 25(1): 1–128. 10.1080/00364827.1966.10409568

[B40] Elofsson R (1970) A presumed new photoreceptor in copepod crustaceans. Cell and Tissue Research 109(3): 316–326. 10.1007/BF02226905 5506526

[B41] Elofsson R (1971) The ultrastructure of a chemoreceptor organ in the head of copepod crustaceans. Acta Zoologica 52(2): 299–315. 10.1111/j.1463-6395.1971.tb00565.x

[B42] Farran GP (1914) Description of a harpacticoid copepod parasitic on an *Octopus*. Annals and Magazine of natural History (Series 8) 13: 472–475. [plate XXI.] 10.1080/00222931408693512

[B43] Farran GP (1926) Biscayan plankton collected during a cruise of H.M.S. ‘Research’, 1900. Part XIV. The Copepoda. Zoological Journal of the Linnean Society 36: 219–310. [plates 5–10.] h 10.1111/j.1096-3642.1926.tb02171.x

[B44] Fleeger JW Shirley TC (1990) Meiofaunal responses to sedimentation from an Alaskan spring bloom. II. Harpacticoid population dynamics. Marine Ecology Progress Series 59: 239–247. 10.3354/meps059239

[B45] Franz DR Friedman I (2002) Effects of a macroalgal mat (*Ulvalactuca*) on estuarine sand flat copepods: An experimental study. Journal of Experimental Marine Biology and Ecology 271(2): 209–226. 10.1016/S0022-0981(02)00045-X

[B46] Frase T Richter S (2020) The brain and the corresponding sense organs in calanoid copepods — Evidence of vestiges of compound eyes. Arthropod Structure & Development 54: e100902. 10.1016/j.asd.2019.100902 31991325

[B47] Frost BW (1974) *Calanusmarshallae*, a new species of calanoid copepod closely allied to the sibling species *C.finmarchicus* and *C.glacialis*. Marine Biology 26(1): 77–99. 10.1007/BF00389089

[B48] Fuentes-Reinés JM Suárez-Morales E (2019) New records of Harpacticoida (Crustacea, Copepoda) from a coastal system of northern Colombia. PANAMJAS – Pan-American. Journal of Aquatic Sciences 14: 100–114.

[B49] Garstang W (1900) Preliminary experiments on the rearing of sea-fish larvæ. Journal of the Marine Biological Association of the United Kingdom 6(1): 70–97. 10.1017/S0025315400072386

[B50] George KH (2004) *Meterorinamagnifica* gen. et sp. nov., a new Idyanthidae (Copepoda, Harpacticoida) from the plateau of the Great Meteor Seamount (Eastern North Atlantic). Meiofauna Marina 13: 95–112.

[B51] George KH (2023) Establishment of a new subfamily of Idyanthidae Lang, 1944 with the description of a new species of *Pseudometeorina* George & Wiest, 2015 (Copepoda, Harpacticoida) from the Eratosthenes Seamount (eastern Mediterranean Sea). Marine Biodiversity 53(3): 1–35. 10.1007/s12526-022-01332-x

[B52] George KH Wiest J (2015) *Pseudometeorinamystica* gen. et sp nov., a new Idyanthidae Lang *incertae sedis* (Copepoda, Harpacticoida) from the Guinea Basin (eastern tropical Atlantic). Marine Biodiversity 45(3): 569–580. 10.1007/s12526-014-0240-5

[B53] Gicklhorn J (1930) Zur Kenntnis der Frontalorgane von *Cyclopsstrenuus* Fischer. Zoologischer Anzeiger 90: 209–216.

[B54] Giesbrecht W (1902) Copepoden. Résultats du Voyage du S.Y. Belgica en 1897–1898–1899 sous le commandement de A. De Gerlache de Gomery. Rapports scientifiques, Zoologie. J.-E. Buschmann, Antwerp, 49 pp. [plates I–XIII.]

[B55] Gmelin JF (1791) Vermes. In: Gmelin JF (Ed.) Caroli a Linné, Systema Naturae per Regna Tria Naturae, secundum Classes, Ordines, Genera, Species, cum Characteribus, Differentiis, Synonymis, Locis.Editio Decima Tertia, Aucta, Reformata. Tomus I. Pars VI. Vermes. G.E. Beer, Lipsiae, 3021–3910.

[B56] Gómez S Fuentes-Reines JM (2017) A new species of *Tisbintra* (Harpacticoida, Tisbidae), and range extension for *Geehydrosomabrevipodum* (Harpacticoida, Cletodidae) from northern Colombia. Caldasia 39(1): 1–12. 10.15446/caldasia.v39n1.64583

[B57] Gómez S Puello-Cruz AC González-Rodríguez B (2004) Three new species of *Tisbe* (Copepoda: Harpacticoida) and a new record with complete redescription of *Tisbemonozota* from north-western Mexico. Cahiers de Biologie Marine 45: 9–47. 10.21411/CBM.A.25DED99C

[B58] Gooding RU (1957) On some Copepoda from Plymouth, mainly associated with invertebrates, including three new species. Journal of the Marine Biological Association of the United Kingdom 36(2): 195–221. 10.1017/S0025315400016714

[B59] Gotto RV (1993) Commensal and parasitic copepods associated with marine invertebrates (and whales). In: Kermack DM, Barnes RSK, Crothers JH (Eds) Synopses of the British Fauna, New Series 46: 1–264. [Field Studies Council, Shrewsbury]

[B60] Gurney R (1927) XXXIII. Report on the Crustacea:—Copepoda (littoral and semi-parasitic). Zoological results of the Cambridge expedition to the Suez Canal, 1924. Transactions of the zoological Society of London 22: 451–577. [figs 108–168, table 35.] 10.1111/j.1096-3642.1927.tb00207.x

[B61] Gurney R (1933) Notes on some Copepoda from Plymouth. Journal of the marine biological Association of the United Kingdom, New Series 19: 299–304. 10.1017/S0025315400055909 18919645

[B62] Haugen E Bristow GA Jansen PA (1998) Parasites of red king crab, *Paralithodescamtschaticus*, from the Varangerfjord area, Northern Norway. Proceedings and Abstracts of the Fourth International Crustacean Congress, Amsterdam, 20–24 July 1998, 192 pp.

[B63] Hesse E (1867) Observations sur les Crustacés rares ou nouveaux des côtes de France (douzième article). Annales des Science naturelles. Zoologie 7(5): 199–216.

[B64] Hicks GRF (1980) Structure of phytal harpacticoid copepod assemblages and the influence of habitat complexity and turbidity. Journal of Experimental Marine Biology and Ecology 44(2): 157–192. 10.1016/0022-0981(80)90151-3

[B65] Hicks GRF (1985) Meiofauna associated with rocky shore algae. In: Moore PG Seed R (Eds) The Ecology of Rocky Coasts, Essays presented to J.R. Lewis, D.Sc. Hodder & Stoughton, London, 36–56.

[B66] Hicks GRF (1988a) Harpacticoid copepods from biogenic substrata in offshore waters of New Zealand. 1: New species of *Paradactylopodia*, Stenhelia (St.) and *Laophonte*. Journal of the Royal Society of New Zealand 18(4): 437–452. 10.1080/03036758.1988.10426467

[B67] Hicks GRF (1988b) Evolutionary implications of swimming behaviour in meiobenthic copepods. In: Boxshall GA Schminke HK (Eds) Biology of Copepods.Proceedings of the Third International Conference on Copepoda. Hydrobiologia 167/168, 497–504. 10.1007/BF00026344

[B68] Hicks GRF Coull BC (1983) The ecology of marine meiobenthic harpacticoid copepods. Annual Review of Oceanography and marine. Biology 21: 67–175.

[B69] Ho J-s (1982) Copepods associated with echinoderms of the Sea of Japan. Report of the Sado marine biological Station. Niigata University 12: 33–61.

[B70] Holmes JMC O’Connor JP (1990) A provisional list of the Harpacticoida (Crustacea: Copepoda) of Ireland. Bulletin – Irish Biogeographical Society 13: 44–130.

[B71] Humes AG (1954) *Tisbecelata* n. sp., a harpacticoid copepod from the mantle cavity of the edible mussel in New Brunswick. Journal of the Fisheries Research Board of Canada 11(6): 816–826. 10.1139/f54-046

[B72] Humes AG (1960) The harpacticoid copepod *Sacodiscus* (= *Unicalteutha*) *ovalis* (C.B. Wilson, 1944) and its copepodid stages. Crustaceana 1(3): 279–294. 10.1163/156854060X00294

[B73] Humes AG (1981) Harpacticoid copepods associated with hermit crabs in the Molluccas. Marine Research in Indonesia 22: 1–19. 10.14203/mri.v22i0.392

[B74] Humes AG Ho J (1969) Harpacticoid copepods of the genera *Porcellidium* and *Paraidya* associated with hermit crabs in Madagascar and Mauritius. Crustaceana 17(2): 113–130. 10.1163/156854068X00016

[B75] Humes AG Voight JR (1997) *Cholidyapolypi* (Copepoda: Harpacticoida: Tisbidae), a parasite of deep-sea octopuses in the North Atlantic and Northeastern Pacific. Ophelia 46(1): 65–81. 10.1080/00785326.1997.10432478

[B76] Huys R (1988) A redescription of the presumed associated *Caligopsyllusprimus* Kunz, 1975 (Harpacticoida, Paramesochridae) with emphasis on its phylogenetic affinity with Apodopsyllus Kunz, 1962. Hydrobiologia 162(1): 3–19. 10.1007/BF00014330

[B77] Huys R (1993) Styracothoracidae (Copepoda: Harpacticoida), a new family from the Philippine deep sea. Journal of Crustacean Biology 13(4): 769–783. 10.2307/1549107

[B78] Huys R (2009) Unresolved cases of type fixation, synonymy and homonymy in harpacticoid copepod nomenclature (Crustacea: Copepoda). Zootaxa 2183(1): 1–99. 10.11646/zootaxa.2183.1.1

[B79] Huys R (2016) Harpacticoid copepods-their symbiotic associations and biogenic substrata: a review. In: Huys R (Ed.) Recent Developments in Systematics and Biodiversity of Symbiotic Copepoda (Crustacea)—A Volume in Celebration of the Career of Prof.Il-Hoi Kim. Zootaxa 4174(1), 448–729. 10.11646/zootaxa.4174.1.28 27811811

[B80] Huys R (2021) John Berkeley James Wells—An appreciation of his contributions to harpacticoid diversity and systematics. In: Huys R (Ed.) Systematics and Taxonomy of Harpacticoid Copepods—A Commemorative Volume in Honour of Prof.John B.J. Wells. Zootaxa 5051(1), 011–040. 10.11646/zootaxa.5051.1.5 34810908

[B81] Huys R Böttger-Schnack R (1994) Taxonomy, biology and phylogeny of Miraciidae (Copepoda: Harpacticoida). Sarsia 79(3): 207–283. 10.1080/00364827.1994.10413559

[B82] Huys R Boxshall GA (1991) Copepod Evolution. The Ray Society, London, 468 pp.

[B83] Huys R Clark PF (2009) Case 3467. Zosimidae Seifried, 2003 (Crustacea, Copepoda, Harpacticoida): Proposed emendation of spelling to Zosimeidae to remove homonymy with Zosiminae Alcock, 1898 (Crustacea, Decapoda, Xanthidae). Bulletin of Zoological Nomenclature 66(1): 24–29. 10.21805/bzn.v66i1.a3

[B84] Huys R Conroy-Dalton S (2000) Generic concepts in the Clytemnestridae (Copepoda, Harpacticoida), revision and revival. Bulletin of the Natural History Museum, London. Zoology: Analysis of Complex Systems, ZACS 66: 1–48.

[B85] Huys R Song SJ (2004) The Ismardiidae Leigh-Sharpe (Copepoda, Harpacticoida*incertae sedis*): Enigmatic as ever? Journal of Crustacean Biology 24(1): 37–53. 10.1651/C-2412

[B86] Huys R Gee JM Moore CG Hamond R (1996) Marine and brackish water harpacticoid copepods. Part 1. In: Barnes RSK, Crothers JH (Eds) Synopses of the British Fauna (New Series) 51: 1–352.

[B87] Innocenti G (2009) Collections of the Natural History Museum, Zoological Section «La Specola» of the University of Florence. XXVII. Crustacea, classes Branchiopoda, Ostracoda and Maxillopoda, subclasses Branchiura and Copepoda. Atti della Società Toscana de Scienze naturali di Pisa. Memorie, Serie B 116: 51–59.

[B88] Itô T (1976) Descriptions and records of marine harpacticoid copepods from Hokkaido. VI. Journal of the Faculty of Sciences, Hokkaido University. Zoology: Analysis of Complex Systems, ZACS 20: 448–567.

[B89] Ivanenko VN Ferrari FD Defaye D Sarradin P-M Sarrazin J (2011) Description, distribution and microhabitats of a new species of *Tisbe* (Copepoda: Harpacticoida: Tisbidae) from a deep-sea hydrothermal vent field at the Mid-Atlantic Ridge (37°N, Lucky Strike). Cahiers de Biologie Marine 52: 89–106. 10.21411/CBM.A.D432FA55

[B90] Jansen PA Mackenzie K Hemmingsen W (1998) Some parasites and commensals of red king crabs, *Paralithodescamtschaticus* (Tilesius), in the Barents Sea. Bulletin of the European Association of Fish Pathologists 18: 46–49.

[B91] Khodami S McArthur JV Blanco-Bercial L Martínez Arbizu P (2017) Molecular phylogeny and revision of copepod orders (Crustacea: Copepoda). Scientific Reports 7(1): e9164. 10.1038/s41598-017-06656-4 PMC5567239 28831035

[B92] Kiko R Michels J Mizdalski E Schnack-Schiel SB Werner I (2008) Living conditions, abundance and composition of the metazoan fauna in surface and sub-ice layers in pack ice of the western Weddell Sea during late spring. Deep-sea Research. Part II, Topical Studies in Oceanography 55(8–9): 1000–1014. 10.1016/j.dsr2.2007.12.012

[B93] Kramer M Swadling KM Meiners KM Kiko R Scheltz A Nicolaus M Werner I (2011) Antarctic sympagic meiofauna in winter: Comparing diversity, abundance and biomass between perennially and seasonally ice-covered regions. Deep-sea Research. Part II, Topical Studies in Oceanography 58(9–10): 1062–1074. 10.1016/j.dsr2.2010.10.029

[B94] Krishnaswamy S (1959) On a new species of *Laophonte* (Copepoda: Harpacticoida) from Madras. Records of the Indian Museum 54(1–2): 29–32. 10.26515/rzsi/v54/i1-2/1956/162006

[B95] Land MF (1978) Animal eyes with mirror optics. Scientific American 239(6): 126–134. 10.1038/scientificamerican1278-126

[B96] Land MF (1984) Crustacea. In: Ali MA (Ed.) Photoreception and Vision in Invertebrates.Plenum Press, New York, 401–438. 10.1007/978-1-4613-2743-1_11

[B97] Land MF (1988) The functions of eye and body movements in *Labidocera* and other copepods. The Journal of Experimental Biology 140(1): 381–391. 10.1242/jeb.140.1.381

[B98] Lang K (1944) Monographie der Harpacticiden (Vorläufige Mitteilung). Almqvist & Wiksells Boktryckeri Ab, Uppsala, 39 pp.

[B99] Lang K (1948) Monographie der Harpacticiden. Håkan Ohlsson, Lund, 1682 pp. [2 vols]

[B100] Leach WE (1816) Annulosa. Supplement to the Fourth, Fifth and Sixth Editions of the Encyclopædia Britannica with Preliminary Dissertations of the History of the Sciences (Vol. 1). Archibald Constable & Co, Edinburgh, 401–453. [plates XX–XXVI.]

[B101] Lee CNW (2004) Distribution of necrophagous copepods in the Cape d’Aguilar Marine Reserve, Hong Kong. Zoological Studies 43: 304–313.

[B102] Lee CNW Morton B (2004) Temporal patterns of change in the necrophagous hyperbenthic zooplankton community of Lobster Bay, Cape d’Aguilar Marine Reserve, Hong Kong. Journal of the Marine Biological Association of the United Kingdom 84(3): 531–538. 10.1017/S0025315404009531h

[B103] Lilljeborg W (1853) Om de inom Skåne Förekommande Crustaceer af Ordingarne Cladocera, Ostracoda och Copepoda. De crustaceis ex Ordinibus Tribus: Cladocera, Ostracoda et Copepoda, in Scania Occurrentibus. Berlingska Boktryckeriet, Lund, 222 pp.

[B104] Linnaeus C (1758) Systema Naturæ per Regna tria Naturæ, Secundum Classes, Ordines, Genera, Species, cum Characteribus, Differentiis, Synonymis, Locis. Tomus I. Editio decima, reformata. Laurentii Salvii, Holmiæ, 824 pp. 10.5962/bhl.title.542

[B105] Loots C Swadling KM Koubbi Ph (2009) Annual cycle of distribution of three ice-associated copepods along the coast near Dumont d’Urville, Terre Adélie (Antarctica). Journal of Marine Systems 78(4): 599–605. 10.1016/j.jmarsys.2009.01.003

[B106] López-González PJ Bresciani J Huys R González AF Guerra A Pascual S (2000) Description of *Genesisvulcanoctopusi* gen. et sp. nov. (Copepoda: Tisbidae) parasitic on a hydrothermal vent octopod and a reinterpretation of the life cycle of cholidyinid harpacticoids. Cahiers de Biologie Marine 41: 241–253. 10.21411/CBM.A.94121E5B

[B107] Makabe R Hasegawa T Sano M Kashiwase H Moteki M (2022) Copepod assemblages in the water column and drifting sea-ice floes in the ice-edge region in the Indian Ocean sector of the Southern Ocean during the austral summer. Polar Biology 45(4): 749–762. 10.1007/s00300-022-03030-7

[B108] Milne Edwards H (1837) Histoire naturelle des Crustacés, comprenant l’Anatomie, la Physiologie et la Classification de ces Animaux. Tome deuxième. Librairie Encyclopédique de Roret, Paris, 531 pp. 10.5962/bhl.title.6234

[B109] Morales-Serna FN Gómez S (2008) First record and redescription of *Tisbellapulchella* (Copepoda: Harpacticoida) from the eastern tropical Pacific. Revista Mexicana de Biodiversidad 79: 103–116. 10.22201/ib.20078706e.2008.001.537

[B110] Moura G Martínez Arbizu P (2003) The phylogenetic position of the bathyal harpacticoids *Aspinothorax* gen. n. and *Styracothorax* Huys (Crustacea: Copepoda). Bulletin de l’Institut royal des Sciences naturelles de Belgique. Biologie 73: 169–184.

[B111] Müller GW (1895) Reports on the dredging operations off the west coast of central America to the Galapagos, to the west coast of Mexico, and in the Gulf of California, in charge of Alexander Agassiz, carried on by the U.S. Fish Commission streamer “Albatross”, during 1891, Lieut. Commander Z.L. Tanner, U.S.N., commanding. XIX. Die Ostracoden. Bulletin of the Museum of Comparative Zoology at Harvard College 27(5): 155–169. [plates I–III.]

[B112] Müller J Troschel FH (1842) System der Asteriden. F. Vieweg & Sohn, Braunschweig, 134 pp. [plates I–XII.] 10.5962/bhl.title.11715

[B113] Nillson D-E (1997) Eye design, vision and invisibility in planktonic invertebrates. In: Lenz PH Hartline DK Purcell JE Macmillan DL (Eds) Zooplankton: Sensory Ecology and Physiology.Gordon & Bearch Publishers, Amsterdam, 149–162. 10.1201/9780203733615-10

[B114] Nishida S Ohtsuka S Parker AR (2002) Functional morphology and food habits of deep-sea copepods of the genus *Cephalophanes* (Calanoida: Phaennidae): perception of bioluminescence as a strategy for food detection. Marine Ecology Progress Series 227: 157–171. 10.3354/meps227157

[B115] Ohtsuka S Hirano K Miyagawa C Kondo Y Sugaya E Nakai T Takada K Fukushima H Oba Y Sambongi Y Asakawa M Nishikawa J (2019) Interactions between marine zooplankters and bacteria (review). Bulletin of the Plankton Society of Japan 66: 86–100. [In Japanese with English abstract] 10.24763/bpsj.66.2_86

[B116] Omori M (1965) A 160-cm opening-closing plankton net: I. Description of the gear. Journal of the Oceanographical Society of Japan 21(5): 212–220. 10.5928/kaiyou1942.21.212

[B117] Paul’son O (1875) Studies on Crustacea of the Red Sea with Notes Regarding Other Seas. Part I. Podophthalmata and Edriophthalmata (Cumacea). S.V. Kulzhenko, Kiev, 144 pp. [22 plates.] [In Russian, English translation by the Israel Program for Scientific Translations, Jerusalem, 1961]

[B118] Reid JW Hribar LJ (2006) Records of some Copepoda (Crustacea) from the Florida Keys. Proceedings. Academy of Natural Sciences of Philadelphia 155(1): 1–7. 10.1635/i0097-3157-155-1-1.1

[B119] Sars GO (1903) CopepodaHarpacticoida. Parts I & II, Misophriidæ, Longipediidæ, Cerviniidæ, Ectinosomidæ (part). An Account of the Crustacea of Norway, with short Descriptions and Figures of all the Species 5: 1–28. [+ plates I–XVI.]

[B120] Sars GO (1904) CopepodaHarpacticoida. Parts V & VI. Harpacticidæ (concluded), Peltidiidæ, Tegastidæ, Porcellidiidæ, Idyidæ (part). An Account of the Crustacea of Norway, with short Descriptions and Figures of all the Species 5: 57–80. [+ plates XXXIII–XLVIII.]

[B121] Sars GO (1905) CopepodaHarpacticoida. Parts VII & VIII. Idyidæ (continued), Thalestridæ (part). An Account of the Crustacea of Norway, with short Descriptions and Figures of all the Species 5: 81–108. [+ plates XLIX–LXIV.]

[B122] Sars GO (1907) Notes supplémentaires sur les Calanoïdés de la Princesse-Alice. (Corrections et additions). Bulletin de l’Institut Océanographique 101: 1–27.

[B123] Sars GO (1909a) Crustacea. Report of the second Norwegian Arctic Expedition in the. Fram 1898–1902(18): 1–47.

[B124] Sars GO (1909b) CopepodaHarpacticoida. Parts XXVII & XXVIII. Cletodidæ (concluded), Anchorabolidæ, Cylindropsyllidæ, Tachidiidæ (part). An Account of the Crustacea of Norway, with short Descriptions and Figures of all the Species 5: 305–336. [+ plates CCIX–CCXXIV.]

[B125] Sars GO (1911) CopepodaHarpacticoida. Parts XXXV & XXXVI. Supplement (concluded), index, etc. An account of the Crustacea of Norway, with short descriptions and figures of all the species 5: 421–449. [+ i–xiv (text) + i–xii (plates) + title of text and of plates + supplement plates 43–54.]

[B126] Schnack-Schiel SB Thomas D Dahms H-U Haas C Mizdalski E (1998) Copepods in Antarctic sea ice. Antarctic sea ice biological process, interactions, and variability. Antarctic Research Series 73: 173–182. 10.1029/AR073p0173

[B127] Schnack-Schiel SB Dieckmann GS Gradinger R Melnikov IA Spindler M Thomas DN (2001a) Meiofauna in sea ice of the Weddell Sea (Antarctica). Polar Biology 24(10): 724–728. 10.1007/s003000100273

[B128] Schnack-Schiel SB Thomas DN Haas C Dieckmann GS Alheit R (2001b) The occurrence of the copepods *Stephoslongipes* (Calanoida) and *Drescheriellaglacialis* (Harpacticoida) in summer sea ice in the Weddell Sea, Antarctica. Antarctic Science 13(2): 150–157. 10.1017/S0954102001000232

[B129] Schnack-Schiel SB Dieckmann GS Kattner G Thomas DN (2004) Copepods in summer platelet ice in the eastern Weddell Sea, Antarctica. Polar Biology 27(8): 502–506. 10.1007/s00300-004-0613-5

[B130] Schnack-Schiel SB Haas C Michels J Mizdalski E Schünemann H Steffens M Thomas DN (2008) Copepods in sea ice of the western Weddell Sea during austral spring 2004. Deep-sea Research. Part II, Topical Studies in Oceanography 55(8–9): 1056–1067. 10.1016/j.dsr2.2007.12.014

[B131] Scott A (1896) Description of new and rare Copepoda. In: Herdman WA (Ed.) Report on the Investigations Carried on in 1895 in Connection with the Lancashire Sea-Fisheries Laboratory at University College, Liverpool.Proceedings and Transactions of the Liverpool biological Society 10: 134–158. [plates I–V. [explanation of plates on 174–177]]

[B132] Scott A (1909) The Copepoda of the Siboga Expedition. Part I. Free-swimming, littoral and semi-parasitic Copepoda. Siboga-Expeditie. Uitkomsten op zoologisch, botanisch, oceanographisch en geologisch gebied verzameld in Nederlandsch Oost-Indië 1899–1900 aan boord H.M. Siboga onder commando van Luitenant ter zee 1^e^ kl. F. Tydeman 29a: 1–323. [plates I–LXIX.] 10.5962/bhl.title.58653

[B133] Scott T (1912) The Entomostraca of the Scottish National Antarctic Expedition, 1902–1904. Transactions of the Royal Society of Edinburgh 48(3): 521–599. 10.1017/S0080456800015829

[B134] Scott T Scott A (1893) Notes on Copepoda from the Firth of Forth: *Longipediacoronata*, Claus; and a preliminary description of an apparently new genus and species. Annals of Scottish natural. History 1893: 89–94. [plate II.]

[B135] Seifried S (2003) Phylogeny of Harpacticoida (Copepoda): Revision of “Maxillipedasphalea” and Exanechentera. Cuvillier Verlag, Göttingen, 259 pp.

[B136] Sewell RBS (1940) Copepoda, Harpacticoida. The John Murray Expedition 1933–1934. Scientific Reports 7(2): 117–382.

[B137] Stebbing TRR (1910) General catalogue of South African Crustacea (Part V. of S. A. Crustacea, for the Marine Investigations in South Africa). Annals of the South African Museum 6(4): 281–599. 10.5962/bhl.part.15558 [plates XV–XXII.] 10.5962/bhl.part.15558

[B138] Steck M Theam KC Porter ML (2023) The cornucopia of copepod eyes: The evolution of extreme visual system novelty. In: Buschbeck E Bok M (Eds) Distributed Vision: From Simple Snsors to Sophisticated Combination Eyes.Springer Series in Vision Research, 223–266. 10.1007/978-3-031-23216-9_9

[B139] Steuer A (1928) Ueber das sogenannte Leuchtorgan des Tiefsee-Copepoden *Cephalophanes* G.O. Sars. Arbeiten aus dem Zoologischen Institut der Universität Innsbruck 3: 9–16.

[B140] Stock JH (1960) Sur quelques Copépodes associés aux invertébrés des côtes du Roussillon. Crustaceana 1(3): 218–257. 10.1163/156854060X00276

[B141] Swadling KM McPhee AD McMinn A (2000) Spatial distribution of copepods in fast ice of eastern Antarctica. Polar Bioscience, Tokyo 13: 55–65.

[B142] Tilesius WL (1815) De cancris camtschaticis, oniscus, entomostracis et cancellis marinis microscopicis noctilucentibus, cum tabulis IV. aeneis et appendice adnexo de acaris et ricinis camtschaticis. Mémoires de l’Académie Impériale des Sciences, St. Pétersbourg 5: 331–405. [plates V–VIII.]

[B143] Ummerkutty ANP (1961) Studies on Indian copepods. 2. An account of the morphology and life history of a harpacticoid copepod, *Tisbintrajonesi*, sp. nov. from the Gulf of Mannar. Journal of the Marine Biological Association of India 2: 149–164.

[B144] Vaissière R (1961) Morphologie et histologie comparée des yeux des Crustacés Copépodes. Archives de Zoologie Expérimentale et Générale 100: 1–125.

[B145] Volkmann B (1979a) A revision of the genus *Tisbella* (Copepoda, Harpacticoida). Archivio di Oceanografia e Limnologia 19(Supplement): 77–119.

[B146] Volkmann B (1979b) A revision of the genus *Tisbe* (Copepoda, Harpacticoida). Part I. Archivio di Oceanografia e Limnologia 19(Supplement): 121–283.

[B147] Wallis JR Swadling KM Everett JD Suthers IM Jones HJ Buchanan PJ Crawford CM James LC Johnson R Meiners KM Virtue P Westwood K Kawaguchi S (2016) Zooplankton abundance and biomass size spectra in the East Antarctic sea-ice zone during the winter-spring transition. Deep-sea Research. Part II, Topical Studies in Oceanography 131: 170–181. 10.1016/j.dsr2.2015.10.002

[B148] Walters K Bell SS (1986) Diel patterns of active vertical migration in seagrass meiofauna. Marine Ecology Progress Series 34: 95–103. 10.3354/meps034095

[B149] Wells JBJ (1970) Copepoda. – I. Sub-order Harpacticoida. Fiches d’Identification du Plancton 133: 1–7.

[B150] Wells JBJ (2007) An annotated checklist and keys to the species of CopepodaHarpacticoida (Crustacea). Zootaxa 1568(1): 1–872. 10.11646/zootaxa.1568.1.1

[B151] Wiborg KF (1964) Marine copepods of Tristan da Cunha. Results of the Norwegian scientific Expedition to Tristan da Cunha, 1937–1938 51: 1–44.

[B152] Willey A (1930) Harpacticoid Copepoda from Bermuda.—Part I. Annals and Magazine of natural History (Series 10) 6: 81–114. 10.1080/00222933008673192

[B153] Wilson CB (1924) New North American parasitic copepods, new hosts and notes on copepod nomenclature. Proceedings of the United States National Museum 64(17): 1–22. 10.5479/si.00963801.64-2507.1

[B154] Wilson CB (1932) The copepods of the Woods Hole region, Massachusetts. Bulletin of the United States national Museum 158: 1–635. [plates 1–41.] 10.5479/si.03629236.158.i

[B155] Wilson CB (1944) Parasitic copepods in the United States National Museum. Proceedings of the United States national Museum 94: 529–582. [plates 20–34.] 10.5479/si.00963801.94-3177.529

[B156] Yeatman HC (1962) The problem of dispersal of marine littoral copepods in the Atlantic Ocean, including some redescriptions of species. Crustaceana 4(4): 253–272. 10.1163/156854062X00238

[B157] Yeatman HC (1963) Some redescriptions and new records of littoral copepods for the Woods Hole, Massachusetts region. Transactions of the American Microscopical Society 82(2): 197–209. 10.2307/3223995

[B158] Yeatman HC (1983) Copepods from microhabitats in Fiji, Western Samoa, and Tonga. Micronesica. Agana 19: 57–90.

[B159] Zwerner DE (1967) *Neoscutellidiumyeatmani* n. g., n. sp. (Copepoda: Harpacticoida) from the Antarctic fish *Rhigophiladearborni* Dewitt, 1962. Transactions of the American Microscopical Society 86(2): 152–157. 10.2307/3224682

